# The Association between the Burden of PM_2.5_-Related Neonatal Preterm Birth and Socio-Demographic Index from 1990 to 2019: A Global Burden Study

**DOI:** 10.3390/ijerph191610068

**Published:** 2022-08-15

**Authors:** Zeyu Tang, Jinzhu Jia

**Affiliations:** 1 Department of Biostatistics, School of Public Health, Peking University, No. 38, Xueyuan Road, Beijing 100871, China; 2 Center for Statistical Science, Peking University, 5 Summer Palace Road, Beijing 100871, China

**Keywords:** premature delivery, fine particulate matter, socio-demographic index, environment, air pollution, disease burden

## Abstract

Background: Preterm birth (PTB) leads to short-term and long-term adverse effects on newborns. Exposure to fine particulate matter (PM_2.5_) was positively related to PTB. However, the global annual average PM_2.5_ was three times than the recommended value in 1998–2014. Socio-demographic index (SDI) is a new indicator that comprehensively reflects the overall development level of a country, partly because of “the epidemiological transition”. Among other countries with higher and similar SDI levels, policy makers have the opportunity to learn from their successful experiences and avoid their mistakes by identifying whether their burdens of disease are higher or lower than the expected. However, it is unclear about the trends of the burden of PM_2.5_-related preterm birth in different countries and different levels of SDI regions. Additionally, the relationship between the SDI and the burden in 1990–2019 is also unclear. Methods: This was a retrospective study based on the Global Burden of Disease Study 2019 (GBD2019) database from 1990 to 2019. The burden of PM_2.5_-related PTB was measured by the age-standardized mortality rate (ASMR), age-standardized disability-adjusted life years rate (ASDR), mortality rate, and the disability-adjusted life years (DALYs). The annual percentage changes (APCs) and the average annual percentage changes (AAPCs) were used to reflect the trends over the past 30 years, which were calculated using a joinpoint model. The relationships between the ASMR, ASDR, and SDI were calculated using a Gaussian process regression. Findings: In 2019, the entire burden of PM_2.5_-related PTB was relatively high, where the ASMR and the ASDR were 0.76 and 67.71, increasing by 7.04% and 7.12%, respectively. It mainly concentrated on early neonates, boys, and on low-middle SDI regions. The increase in the burden of PM_2.5_-related PTB in low and low-middle SDI regions is slightly higher than the decrease in other SDI regions. In 2019, the burden varied greatly among different levels of SDI regions where ASMRs varied from 0.13 in high SDI regions to 1.19 in low-middle regions. The relationship between the expected value of the burden of PM_2.5_-related PTB and SDI presented an inverted U-shape, and it reached the maximum when SDI is around 0.50. The burdens in four regions (South Asia, North Africa and the Middle East, western sub-Saharan Africa, and southern sub-Saharan Africa) were much higher than the mean value. Boys bore more burden that girls. The sex ratio (boys:girls) of the burden showed a dramatically increasing trend in low SDI regions and a decreasing trend in middle SDI regions and high-middle SDI regions. These differences reflect the huge inequality among regions, countries, ages, and sex in the burden of PM_2.5_-related PTB. Conclusion: The overall burden of PM_2.5_-related PTB in 2019 was relatively high, mainly concentrated on early neonates, boys, and on low-middle SDI regions. It showed an increasing trend in low-middle and low SDI regions. The association between the burden and the SDI presented an inverted U-shape. It is very necessary to promulgate policies to prevent and control air pollution in countries with large and increasing exposure to PM_2.5_ pollution because it does not need action at an individual level. Focusing on public educational interventions, public and professional policies, and improving accessibility of prenatal care are other feasible ways for low and low-middle SDI countries. Policy makers should also appropriately allocate medical resources to boys and early newborns.

## 1. Introduction

Preterm birth is defined as delivery less than 37 weeks or less than 259 days from the first day of a pregnant woman’s last menstruation to delivery [1]. In the world, there were approximately 14.9 million preterm birth babies, accounting for 11.1% of all live births [2]. Its incidence rate in all livebirths varied greatly, ranging from approximately 5% in some Europe countries to 18% in several Asia countries [2]. Preterm birth was one of the main causes of death in children under 5 years old, accounting for 14.1% [3]. In the long run, it can also cause a series of adverse outcomes related to the neural development, including cerebral palsy, cognitive impairment, weak motor coordination, and learning difficulties [4]. Preterm births brought high economic cost, for preterm babies need intensive care in the short term, as well as health care and educational support in long term. For example, it brought about a social burden of about USD 26 billion to the United States in 2005 [5].

Some risk factors are related to preterm birth, including ethnic group (black, African-American, Afro-Caribbean) [6,7], low socioeconomic and educational status, maternal ages (low and high), single marital status [8,9,10], short inter-pregnancy interval [11], pre-pregnancy body-mass index (low and high) [12,13,14], having a previous preterm delivery [15], multiple gestations [16], and high levels of psychological or social stress [17,18]. Some studies found that PM_2.5_ was positively associated with pre-term birth in the developing and developed countries. Bekkar et al. conducted a large systematic review where 24 studies, including 318,960 births, evaluated the association between maternal exposure to PM_2.5_ and preterm birth [19]. They found that there were positive associations in all geographic regions of the United States and there were positive associations between exposure to PM_2.5_ and the increased risk of preterm birth in 19 of 24 studies (79%). Based on the 11 studies evaluating whole-pregnancy exposure to PM_2.5_, the risk increased and its median was 11.5% (2–19%). Ottone et.al found that preterm birth was associated with PM_2.5_ (OR: 1.03, 95% Confidence Interval: 1.002–1.058 per 1 μg/m^3^) using three-year measurements of daily concentrations of PM_2.5_ in northern Italy [20]. Bachwenkizi et al. also found that the odds ratios reached 1.08 (95% confidence interval: 1.01–1.16) in Africa with an interquartile range (33.9-μg/m^3^) increase in PM_2.5_ [21]. In addition, a study conducted in 336 cities of China showed that the risk of preterm birth increased by 7% for each interquartile range’s (29-μg/m^3^) increase in exposure of PM_2.5_ [22]. There are some mechanisms that may explain how PM_2.5_ leads to premature birth. PM_2.5_ could affect transplacental oxygen and nutrient transport and eventually leads to preterm birth through some complex mechanisms, such as oxidative stress, pulmonary and placental inflammation, blood coagulation, endothelial function, and hemodynamic responses [23]. These mechanisms may be interrelated because oxidative or nitrosative stress may produce adverse effects and lead to dysfunction of the placenta vascular. In addition, Brook et al. pointed out that diastolic blood pressure could be increased by PM_2.5_ through inducing acute autonomic imbalance, and it was the explanation for vascular dysfunction [24].

Socio-demographic index (SDI) is a new indicator that comprehensively reflects the overall development level of a country. It was first proposed by Global Burden of Disease (GBD) researchers in 2015 and it consists of key factors—a country’s income per capita, average level of educational attainment, and fertility rate—contributing to the health level of the population [25]. The research team compared the disease burden between countries with different SDI levels, rather than between developing and developed countries, because of “the epidemiological transition”; that is, health problems in developing countries were increasingly similar to that in developed countries [26]. In this transition, a population tends to have longer life expectancy, lower mortality rates because of infectious diseases but higher mortality rates caused by non-infectious diseases (for example, diabetes), and higher age-related disability rates. Due to these countries tending to be similar in this way, it is of little sense to evaluate the difference of disease burden between developed and developing countries [26]. Using SDI, researchers can further study the relationship between the disease burden and the socio-economic core factors contributing to population health and provide reasonable suggestions for policymakers. Some high-level studies used SDI to evaluate the burden of disease [27,28,29]. For instance, Liu et al. evaluated the global burden of PM_2.5_-related type 2 diabetes in 1990–2019.

PM_2.5_ showed different trends over time in different SDI regions. The average annual PM_2.5_ concentration weighted by the population in the world was twice as high as the value suggested by the World Health Organization in 1998–2014. This was mainly due to the sharp rise in PM_2.5_ concentrations in Asia and Africa, most of which are at the level of low and middle socio demographic index [30]. However, the changes of annual PM_2.5_ concentrations showed negative trends in some regions with high-middle and high SDI levels, including eastern North America (−0.28 ± 0.03 μg/m^3^/yr) and Europe (−0.15 ± 0.03 μg/m^3^/yr), in 1998–2018. Remarkably, there was a negative trend in China (−3.37 ± 0.38 μg/m^3^/yr, a middle SDI country) over 2011–2018 [31].

Some previous studies have evaluated the burden of preterm birth in the world from different aspects. Chawanpaiboon et.al estimated incidence rates of preterm birth in 2014 and its changing trends in some countries [32]. Malley et al. firstly evaluated the number and rate of PM_2.5_-associated preterm births in the world in 2010. It was estimated as 2.7 million (18%) and 3.4 million (23%) with low concentration cut-off (LCC) set at 10 μg/m^3^ and 4.3 μg/m^3^, respectively [33]. Cohen et al. further evaluated the burden of preterm birth attributed to PM_2.5_ in 2019 and its trends over the past 25 years, measured by disability-adjusted life years, years of life lost because of premature mortality, and years lived with a disability [34].

However, it is unclear about the trends of PM_2.5_-related preterm birth burden in regions and countries with different levels of SDI. Additionally, it is also unclear about the relationship between the burden and SDI over the past 30 years. To fill these gaps, the purposes of this study are to: (1) evaluate the burden of PM_2.5_-related preterm birth and its trend in the world from 1990–2019; (2) evaluate the relationship between the SDI and the burden of PM_2.5_-related preterm birth from 1990 to 2019; (3) evaluate the PM_2.5_-related preterm birth burden in different age groups and by males and females; (4) analyze the possible explanations for related trends and provide valuable advice to policy makers.

## 2. Methods

### 2.1. Data

We used the Global Health Data Exchange to obtain data from Global Burden of Disease Study 2019 (GBD 2019) [35,36,37]. The GBD 2019 is a high-quality data set and the missing values had been carefully and skillfully processed [36]. Some high level studies used GBD 2019 data [29,35,37]. The data used age-standardized mortality rates (ASMR, per 100,000 population), age-standardized disability-adjusted life year rates (ASDR, per 100,000 population), and disability-adjusted life years (DALYs) as main indicators to measure the disease burden. PM_2.5_ values, causes, risk factors, different age group, sex, and SDI are also available in the data, covering 192 countries and regions in 1990–2019.

ASMR is defined as a weighted average of the age-specific mortality rates per 100,000 persons, where weights are defined as the proportions of persons in the corresponding age groups of the World Health Organization (WHO) standard population. ASDR is calculated by weighting DALYs according to different age distribution in the population. DALYs is defined as the total years of life lost due to premature mortality (YLLs) and the years lived with a disability (YLDs) due to related cases of the disease or health condition in a population. The YLLs are lost years due to early death, while the YLDs are any short-term or long-term healthy life years lost as a result of disability. All the definitions mentioned above are available at the WHO website (www.who.int/data/gho/indicator-metadata-registry (accessed on 4 January 2022)). According to the International Classification of Diseases, Tenth Revision (ICD-10), neonatal preterm birth in the GBD 2017 was defined by codes P07.2–P07.39, P22–P22.9, P25–P28.9, P61.2, P77–P77.9 [38]. Early and late neonatal period are defined as age 0–6 days and 7–27 days, respectively [39].

The calculation of ASMR and ASDR of preterm birth, attributable to PM_2.5_, is based on the estimation of population-weighted exposure to PM_2.5,_ theoretical minimum risk exposure level (TMREL), estimation of the distribution of exposure for PM_2.5_, the estimation of the deaths and DALYs of neonatal preterm births attributable to PM_2.__5_, and the estimation of the ASMR and ASDR of preterm birth attributable to PM_2.__5_ [34,36].

The PM_2.5_ values were obtained from ground measurements and satellite retrievals. Annual average fine particle (PM_2.5_) was calculated by the combination of PM_2.5_ values and chemical transport model simulations at the resolution of 11 km × 11 km. Using these estimates, population-weighted mean concentrations can be calculated and then used to calculate the relative risk of disease mortality with the basis of integrated exposure–response functions for each cause of death [34,40].

The TMREL of PM_2.5_ was assumed to be a uniform distribution of 2.4–5.9 µg/m^3^. The lower and upper bounds were calculated from the minimum and fifth percentiles of the distribution of exposure to PM_2.5_ from cohort studies. The uncertainty in this low-level exposure to PM_2.5_ can be represented by a uniform distribution [41,42,43].

Integrated exposure–response functions (IERs) were developed for the death attributable to neonatal preterm birth. The relative risk of mortality was calculated using risk estimates from studies of ambient air pollution [42,44].

Deaths and DALYs due to PM_2.5_ were calculated using the age-specific, year-specific, sex-specific, and location-specific PAF to the numbers of deaths and DALYs [25,45].

$$PAF_{joasgt}=\frac{\int_{x=l}^{u}RR_{joasg}(x)\cdot P_{jasgt}(x)dx-RR_{joasg}(TMREL_{jas})}{\int_{x=l}^{u}RR_{joasg}(x)\cdot P_{jasgt}(x)dx}$$

where $PAF_{joasgt}$ represents the PAF for cause o, for age group a, sex s, location g, and year t; $RR_{joasg}(x)$ represents the relative risk as a function of exposure level, x, for risk factor j, for cause o controlled for confounding, age group a, sex s, and location g, with the lowest level of observed exposure as l and the highest as u; $P_{jasgt}(x)$ represents the distribution of exposure at x for age group a, sex s, location g, and year t; $TMREL_{jas}$ represents the TMREL for risk factor j, age group a, and sex s.

ASMR and ASDR of preterm birth attributable to PM_2.5_ were calculated by weighting the deaths and DALYs of preterm birth attributable to PM_2.5_ according to different age distribution in the population.

As mentioned in the introduction, SDI is calculated by simple factors but can powerfully reflect the overall development of the society, ranging from 0.0 to 1.0. Getting the SDI with 1.0 means a country’s income per capita, average level of educational attainment, and fertility rates are at the highest level in the world [46]. In contrast, a lower SDI means a country is at a lower level of social development.

The division of SDI was initially purposed by GBD 2015 Mortality and Causes of Death Collaborators. They divided geographies into SDI quintiles in 2015. After excluding populations less than 1 million, quintile cutoffs were selected on the basis of entire distribution of geography–years from 1980 to 2015. In 2019, they updated SDI quintiles and divided the world into five regions according to the SDI: low SDI regions (0.000–0.455), low-middle SDI regions (0.455–0.608), middle SDI regions (0.608–0.690), high-middle SDI regions (0.690–0.805), and high SDI regions (0.805–1.000). This definition can be found at the document “SDI Reference Quintiles” in the following link: https://ghdx.healthdata.org/record/ihme-data/gbd-2019-socio-demographic-index-sdi-1950-2019 (accessed on 4 January 2022).

### 2.2. Statistics

We used the ASMR and ASDR of preterm births attributable to PM_2.5_ to reflect the burden of PM_2.5_-related PTB because of the difference of the age structure among countries and regions. We used a joinpoint model to evaluate the percentage change, annual percentage change (APCs), and average annual percentage change (AAPCs) of the burden, where each country or region was set to have 2 knots. APC and AAPC were used to evaluate the change trend of each part and the global change trend in a linear way, respectively. They are two important indicators reflecting the trend of disease burden measured by ASMR and ASDR in 1990–2019 [35]. The calculation of APCs and AAPCs is listed as below:

(1) $$\ln\left(\mathrm{ASMR}\;\mathrm{or}\;\mathrm{ASDR}\right)=\alpha +\beta_{i}x$$

(2) $$\mathrm{APCs}=100\times \left(\exp\left(\beta_{i}\right)-1\right)$$

(3) $$\mathrm{AAPCs}=\left\{\exp\left(\sum w_{i}\beta_{i}/\sum w_{i}\right)-1\right\}\times 100$$

where x represents the year, $\beta_{i}$ represents the slope in each part divided by knots, and $w_{i}$ represents the number of years in each part.

We evaluated the relationship between the burden of PM_2.5_-related PTB and SDI in two ways. First, we used Pearson correlation coefficients to measure the trend between ASMR, ASDR, and SDI at different ranges of SDI (less than 0.44, between 0.44 and 0.60, more than 0.60). Second, the expected value of the disease burden was measured by the Gaussian process regression [47]. It had been used to analyzed trends in some studies [35,48]. The details are as follows.

We assumed that there was a certain link between disease burden among regions with different SDI. This connection was related to the gap of indexes. The smaller the gap, the stronger the connection. Gaussian process has a priori assumption that certain information will be transmitted between adjacent samples, that is, there is a certain correlation between them. This method assumes that the observed variables have the Gaussian distribution, and the relationship between the observed variables is reflected by the kernel matrix.

The observation can be expressed as

(4) $$y_{i}=t\left(x_{i}\right)+\varepsilon_{i}$$

where $y_{i}$ represents the burden of PM_2.5_-related preterm birth, $x_{i}$ is the socio-demographic index, $\varepsilon_{i}$ is an independent Gaussian distribution of which the mean is 0.

The posterior distribution can be expressed as

(5) $$p\left(y|t\right)=\left[\underset{i}{\prod }p\left(y_{i}-t\left(x_{i}\right)\right)\right]\frac{1}{\sqrt{{\left(2\pi \right)}^{m}det\left(K\right)}}exp\left(\frac{1}{2}t^{T}K^{-1}t\right)$$

After substituting t=Kα, we can get

(6) $$ln\left(p|y\right)=-\frac{1}{2\sigma^{2}}\|y-K\alpha {\|}^{2}-\frac{1}{2}\alpha^{T}K\alpha +c$$

where K is the kernel to be selected. In this paper, we chose the Gaussian radial basis function:

(7) $$K\left(x,x^{\prime }\right)=\exp\left(-\sigma \|x-x^{\prime }{\|}^{2}\right)$$

This is a common choice, which is applicable the situation where the data have no prior information.

We used R software [49] and joinpoint model [50,51] to do all analysis and to get pictures. R package “kernlab” was used to develop Gaussian process regression model with gausspr = “rbfdot” [52]. How to conduct Gaussian process regression in R and its mathematical details can be found at vignettes of the R package “kernlab”. The R packages “maps” [53] and “ggplot2” [54] were used to get figures.

## 3. Results

### 3.1. Global Burden of PM_2.5_-Related Preterm Birth in 1990–2019

We studied the distribution and the trends of ASMR and ASDR in 192 countries or regions in the past 30 years. In 2019, the overall burden of PM_2.5_-related preterm birth was relatively high. As shown in Table 1 and Table 2, ASMR was 0.76 in 2019 and ASDR was 67.71. From 1990 to 2019, the ASMR and ASDR increased by 7.04% and 7.12%, respectively, and their AAPCs were 0.2 (95% confidence interval: 0.0, 0.4).

The highest ASMR and ASDR of PM_2.5_-related preterm birth occurred in South Asia (1.49 and 132.13). The second and the third countries were southern sub-Saharan Africa (1.22 and 108.68) and western sub-Saharan Africa (1.13 and 100.06), respectively, however, high-income Asia Pacific had the lowest values (0.04 and 3.74). Western sub-Saharan Africa had the largest increase rate in the ASMR and ASDR (113.21% and 110.39%), while Eastern Europe had the largest decline rate (−79.53% and −36.94%).

Over past thirty years, among twenty-one regions in our analysis, thirteen of them showed a decreasing trend, with the average annual percentage changes in the ASMR varying from −1.4 (95% confidence interval: −1.6, −1.2) in Southeast Asia to −5.6 (95% confidence interval: −7.9, −3.2) in Eastern Europe. In contrast, the AAPCs in the ASMR remained rising in other eight regions ranging from 0.3 (95% confidence interval: 0.0, 0.7) in Central Asia to 2.7 (95% confidence interval: 2.3, 3.0) in western sub-Saharan Africa. A similar trend can be seen in the average annual percentage changes in the ASDR (Table 1 and Table 2). The highest value was found in western sub-Saharan Africa, 2.7 (95% confidence interval: 2.3, 3.0), while the lowest value was found in Eastern Europe at −5.6 (95% confidence interval: −6.8, −4.5). Three-part APCs in the ASMR and ASDR are shown in Table 1 and Table 2. More details about APCs are shown in the Supplementary Materials (Figures S1–S42). 

### 3.2. PM_2.5_-Related Preterm Birth Burden by Socio-Demographic Index Regions

**Figure 1:**
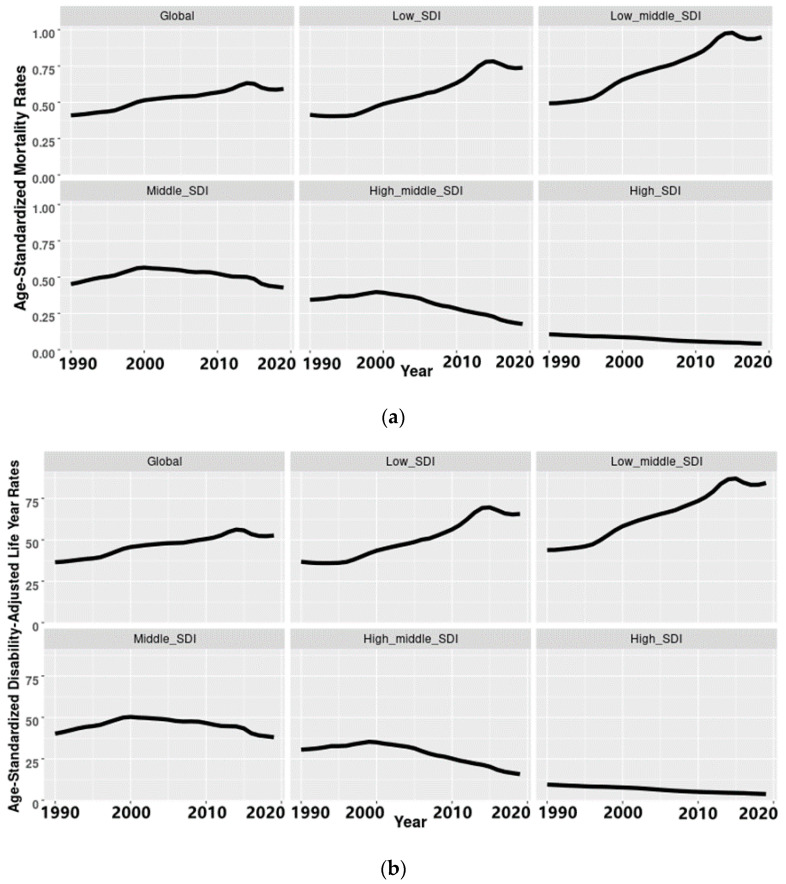
(**a**) The changes of age-standardized mortality rates in PM_2.5_-related preterm birth in the world and different social-demographic index (SDI) regions in 1990–2019. (**b**) The changes of age-standardized disability-adjusted life year rates in PM_2.5_-related preterm birth in the world and different social-demographic index (SDI) regions in 1990–2019.

In 1990–2019, the burden of PM_2.5_-related preterm birth had been increasing in the world, and its increase rates in low and low-middle SDI regions were particularly obvious (Figure 1a,b).

Among all regions with different levels of SDI, the highest ASMR was found in low-middle SDI regions in 2019, 1.19 (95% confidence interval: 0.81, 1.65), and the largest increase in it in the past 30 years, by 67.61% (Table 1). Followed by low-middle SDI regions, the ASMR and its rate of increase were the second and third highest in middle SDI regions and low SDI regions, respectively. On the contrary, the lowest ASMR in 2019 and the greatest decrease in it were found in high SDI regions, 0.13 (95% confidence interval: 0.11, 0.16) and 62.86%, respectively (Table 1).

As seen in Figure 1a, low SDI regions and low-middle SDI regions showed an obvious increasing trend in the ASMR in PM_2.5_-related preterm birth in 1990–2019, where the AAPCs were 1.7 (1.1, 2.2) and 1.9 (1.6, 2.2), respectively. Over the past 30 years, the ASMR has maintained a downtrend in middle, high-middle, and high SDI regions, with average annual percentage changes ranging from −0.7 to −3.2. Similar trends were found in ASDR (Figure 1b).

**Figure 2:**
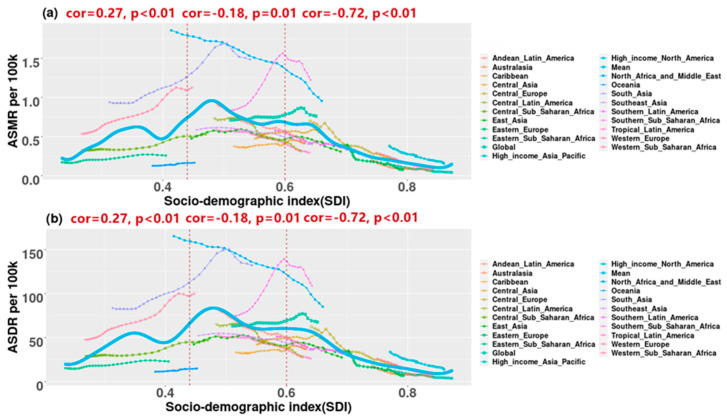
The trends between global and regional burden of PM_2.5_-related preterm birth and socio-demographic index (SDI) from 1990 to 2019: (**a**) age-standardized mortality rates (ASMR); (**b**) age-standardized disability-adjusted life years rates (ASDR). The U-shape solid blue line represents the expected mean value of the burden. Pearson correlation coefficient and *p*-value are used to reflect the trends in each part.

The relationship between the burden of PM_2.5_-related preterm birth and the SDI is shown in Figure 2. The solid blue line represents the expected value of the burden of PM_2.5_-related preterm birth, that is, a country’s SDI is expected to match its burdens. The relationship between SDI and the expected value of the burden presented an inverted U-shape. The expected ASMR in the PM_2.5_-related preterm birth increase gradually with the increase in SDI. It reaches the maximum when SDI is around 0.50, and then decreases gradually with the decrease in SDI. Specifically, between ASMR and SDI, there was moderately positive correlations with SDI < 0.44 and slightly negative correlations with SDI among 0.44–0.60 but strongly negative correlations with SDI > 0.60. The ASMRs in four regions (southern sub-Saharan Africa, South Asia, North Africa and the Middle East, and western sub-Saharan Africa) were much higher than the expected mean value. The trends mentioned above in ASDR are similar to those in ASMR.

### 3.3. The Burden of PM_2.5_-Related Preterm Birth in Different Age and Sex Groups

**Figure 3:**
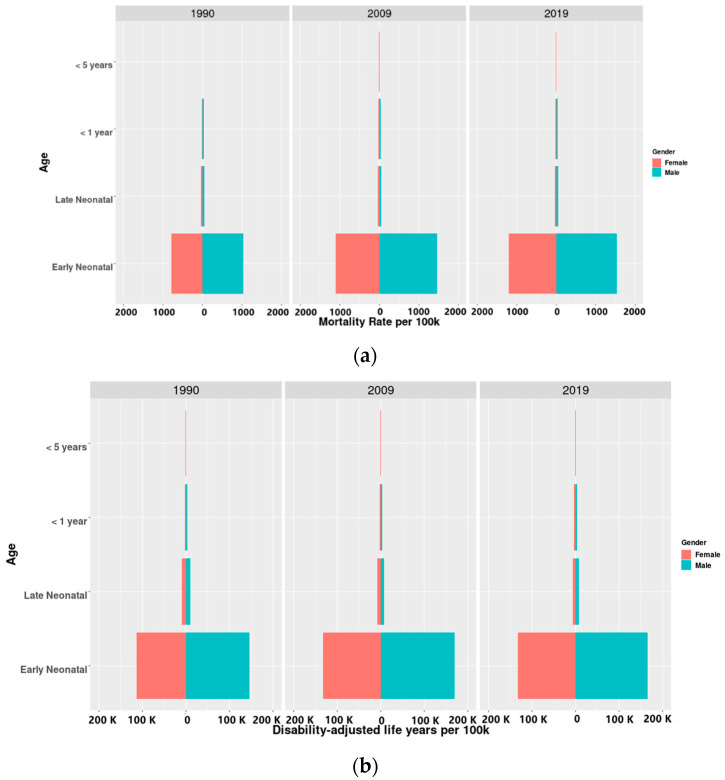
(**a**) Mortality rates (per 100,000 people) of PM_2.5_-related preterm birth in the world in different sex and age groups in 1990, 2009, and 2019. (**b**) Disability-adjusted life years rates (per 100,000 people) of PM_2.5_-related preterm birth in the world in different sex and age groups in 1990, 2009, and 2019.

Figure 3a,b show the trends of the burden of PM_2.5_-related preterm birth by different sex and age groups. The burden of PM_2.5_-related preterm birth is mainly concentrated on early neonates, followed by late neonates. There is almost no burden in other age groups. Boys had higher the burden of PM_2.5_-related preterm birth than girls in 1990, 2009, and 2019. 

**Figure 4:**
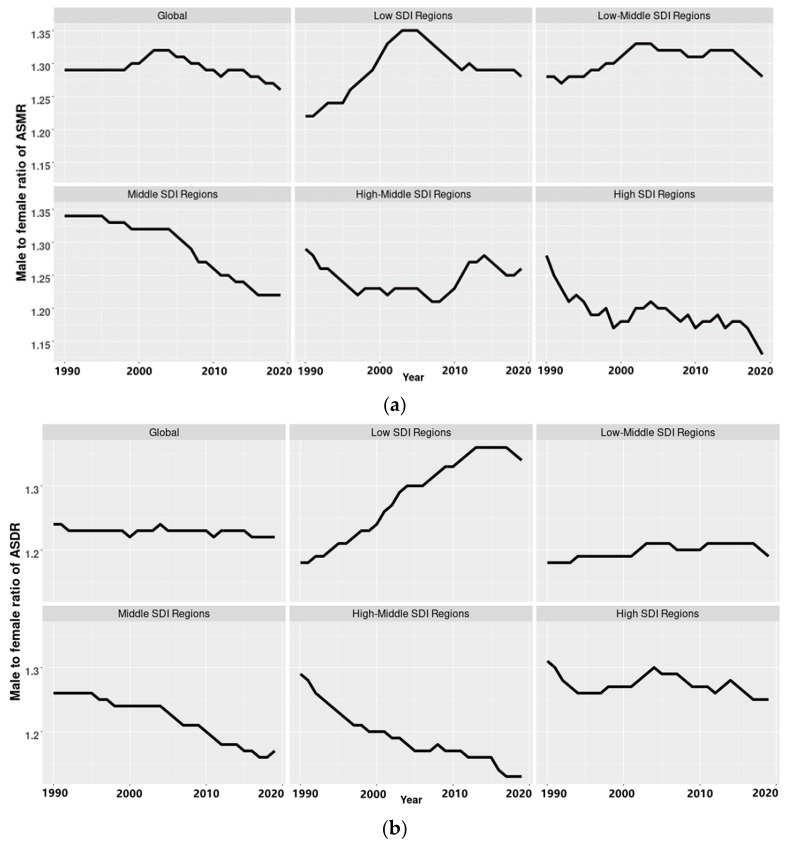
(**a**) The ratio of age-standardized mortality rates (ASMR) in males to that in females in the world and regions with different levels of socio-demographic index (SDI) from 1990 to 2019. (**b**) The ratio of age-standardized disability-adjusted life years rate (ASDR) in males to that in females in the world and regions with different levels of socio-demographic index (SDI) from 1990 to 2019.

Figure 4a,b show the trends of the sex ratio of the burden of PM_2.5_-related preterm birth. The sex ratio of ASMR sharply increased from less than 1.20 to more than 1.35 in the low SDI regions and slightly increased in the low middle regions. It showed a decreasing trend in middle SDI regions and high-middle SDI regions, from around 1.30 to around 1.15. The sex ratio of ASMR fluctuated in high SDI regions, but it was generally high, ranging from about 1.25 to 1.3. Similar trends were seen in the sex ratio of ASDR.

### 3.4. The Burden of PM_2.5_-Related Preterm Birth by Countries

**Figure 5:**
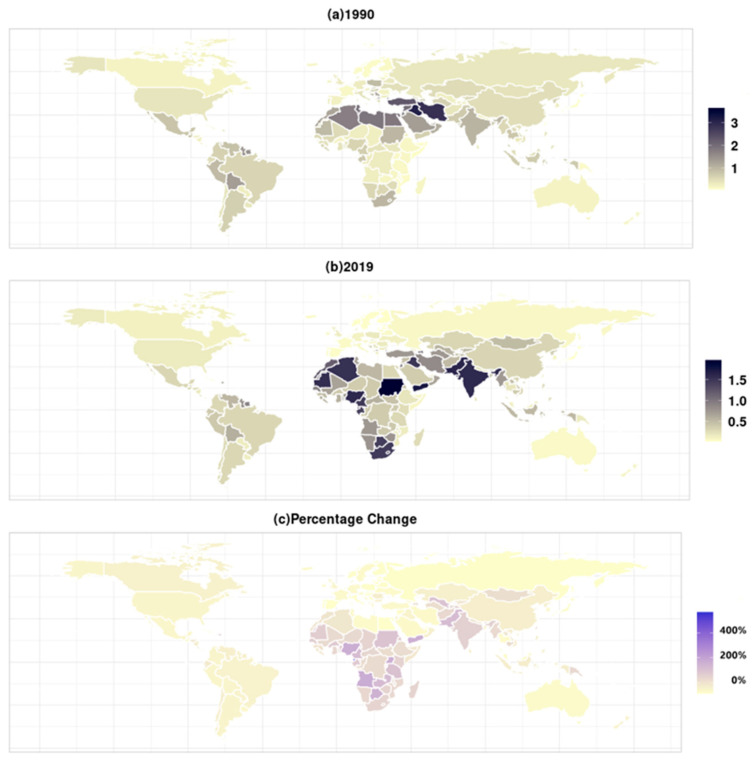
The global age-standardized mortality rates (per 100,000 people) and its percentage changes in PM_2.5_-related preterm birth: (**a**) 1990; (**b**) 2019; (**c**) percentage changes from 1990 to 2019.

Figure 5 showed that the burden of PM_2.5_-related preterm birth varied greatly from country to country. In 2019, the gap between countries with the lowest burden and countries with the highest burden exceeded 90 times. Six countries (Finland, Estonia, Japan, Latvia, Monaco, and Norway) had the lowest ASMR (0.02) in PM_2.5_-related preterm birth, while Sudan had the highest ASMR (1.96). The ASMRs in eight countries (Algeria, Equatorial Guinea, India, Mauritania, Nigeria, Pakistan, Sudan, Yemen) were more than 1.5, however, they were below 0.5 in 139 countries. As shown in Table 1 and Table 2, compared to other regions, four regions (North Africa and Middle East, South Asia, southern sub-Saharan Africa, western sub-Saharan Africa) had a higher burden of PM_2.5_-related preterm birth. Conversely, seven regions (Western Europe, Central Europe, high-income Asia Pacific, Australasia, Oceania, Eastern Europe, high-income North America) had relatively low burden.

From 1990 to 2019, ASMR in PM_2.5_-related preterm birth decreased the most in twenty-four countries, by more than 80%. In contrast, eight countries (Equatorial Guinea, Angola, Botswana, Djibouti, Fiji, Nigeria, Uganda, Yemen) had the highest growth rates, more than 150% (Figure 5). The regions with the lower burden of PM_2.5_-related preterm birth were Central Europe, Australasia, Eastern Europe, Western Europe, high-income Asia Pacific, while the regions with increased burden were the South Asia, central sub-Saharan Africa, eastern sub-Saharan Africa, western sub-Saharan Africa.

## 4. Discussion

We evaluated the burden of PM_2.5_-related preterm birth and its trend in 1990–2019 in the world, regions divided by geography and SDI, 192 countries, age groups and sex groups. We also evaluated the relationship between the burden of PM_2.5_-related preterm birth and the SDI from 1990 to 2019. The overall burden of PM_2.5_-related preterm birth in 2019 was relatively high, mainly concentrated on early neonates, boys, and on low-middle SDI regions. Over the past 30 years, the increase in the burden of the PM_2.5_-related preterm birth in low-middle SDI regions and low SDI regions is slightly higher than the decrease in and high, high-middle, and middle SDI regions. The burden varies greatly among different SDI regions, with ASMRs varying from 0.13 in high SDI regions to 1.19 in low-middle SDI regions in 2019. The relationship between the expected value of the burden of PM_2.5_-related preterm birth, and the SDI presented an inverted U-shape. With the increase in SDI, the burden increased gradually, reaching the maximum when SDI is around 0.50, and then it decreased gradually. The burdens in four regions (southern sub-Saharan Africa, North Africa and Middle East, South Asia, and western sub-Saharan Africa) were much higher than the expected mean value. These four regions should pay more attention on the burden of PM_2.5_-related preterm birth. Boys bore more burden that girls and the sex ratio (boys:girls) of the burden showed a dramatically increasing trend in low SDI regions and showed a decreasing trend in middle SDI regions and high-middle SDI regions. These differences reflect the huge inequality among regions, countries, ages, and sex in the burden of PM_2.5_-related preterm birth.

Over the past 30 years, the burden of PM_2.5_-related preterm birth increased moderately in the world by around 7%. However, it is not because each of the SDI regions increased slightly, instead, the increase in the burden of PM_2.5_-related preterm birth in low-middle and low SDI regions is slightly higher than the decrease in high, high-middle, and middle SDI regions. Some reasons may explain that the burden continued to increase in the low and low-middle SDI regions and continued to decrease in the middle, high-middle, and high SDI regions. First, air quality was mainly improving in high-middle and high SDI countries. For example, the annual change of PM_2.5_ concentrations showed negative trends in some regions at high-middle and high SDI levels, including eastern North America (−0.28 ± 0.03 μg/m^3^/yr) and Europe (−0.15 ± 0.03 μg/m^3^/yr) in 1998–2018 [31]. These improvements were due to the management of air quality [55,56]. In contrast, it was decreasing in some low-middle and low SDI countries. India had positive trends in annual PM_2.5_ concentrations, more than 1 μg/m^3^/yr, and Middle East, central and southern Africa also had positive trends with 0.25–0.50 μg/m^3^/yr [31]. Second, the progress in national policy (such as GDP investment, public health policy, and social provision) and health-care services (such as neonatal intensive care and timely referral) in high-middle and high regions were important to reduce the burden [57,58].

We found that the relationship between the expected value of the burden of PM_2.5_-related preterm birth and the SDI presented an inverted U-shape. With the increase in SDI, the burden increased gradually, reaching the maximum when the SDI is around 0.50, and then it decreased gradually. Some possible reasons may explain that higher SDI regions had a higher burden of PM_2.5_-related preterm birth than lower SDI regions. First, countries with higher SDI levels paid more attention to the improvement of health level and environment protection level [59], and thus the PM_2.5_ pollution levels were reduced [34]. However, the PM_2.5_ exposure level in lower SDI regions is still higher than that in higher SDI regions, especially household air pollution exposure [36]. Second, poverty is the essential reason in causing neonatal deaths. Poor countries had low coverage rates of health care and high prevalence rate of related risk factors, such as maternal infection [60]. There was a huge gap between the richest and poorest 20% of Canadians in neonatal adverse outcomes [61]. Similar results were also found in some sub-Saharan African and South Asian countries [60]. The third reason is the difference of the coverage rate of health-care, policy, and programs support among different levels of SDI regions. In the world, the range of the proportion of women who delivered with skilled care varied from 5 percent to 99 percent [62]. However, lower SDI countries have low rates of accessibility to skilled care and institutional delivery [60]. For example, in sub-Saharan Africa, no more than 40 percent women delivered with skilled care, and this figure was no more than 30 percent in South Asia. Approximately 75% of newborns died in their early neonatal stage and focusing on the cause of neonatal death in the first week after birth is very important to reduce the number of deaths. It is helpful to reduce neonatal mortality rates in poor countries by improving the coverage of care during the early neonatal period [60]. Fourth, timely referral is an important way to save ill babies’ lives. Only 21 percent ill babies in Uganda could get access to the referral [63]. However, compared to the low SDI regions, middle and low-middle SDI regions had a higher burden of PM_2.5_-related preterm birth. It may be explained by the fact that the negative impact of environmental pollution on residents is higher than that of economic development, facility-based delivery, and health care in middle and low-middle SDI regions.

SDI is a new indicator that comprehensively reflects the overall development level of a country. Rather than comparing the difference of disease burden between developing countries and developed countries, it is better to compare them among different SDI countries, which can more deeply reveal the relationship between core social-demographic factors and disease burden, as well as the trend of the distribution of risk factors with the social-demographic index. Policymakers in low, low-middle, and middle SDI countries can learn from the experience in two ways. First, learn from the successful ways of high-middle and high SDI countries to deal with risk factors and disease burden. Second, among countries with similar SDI to theirs, identify countries with higher and lower disease burden than the expected, and then learn from their successful experiences and avoid their mistakes. Specifically, policymakers can learn from the experience of high-middle and high SDI countries in the following aspects: public educational interventions, public and professional policies, and prenatal care. First, the public has an inaccurate understanding that improving the level of care for newborns has solved the problems caused by premature birth [64]. In fact, raising public awareness of potential risk factors and avoiding them is a feasible way [65]. For example, the choices of the public may be affected by publicizing the fact that single pregnancies using assisted reproductive technology may face a higher risk of preterm birth [66,67]. Second, compared with the public educational interventions, the policies promulgated by the government can have a more direct impact [68]. For example, Europe, Australia and the United States have issued policies aimed at reducing the risk of higher-order multiple gestation and achieved good results. From 1996 to 2003, the incidence of higher-order multiple pregnancies decreased by 50% [69,70]. As another example, the vast majority of European countries adopted social security policies to improve pregnancy outcomes, such as paid maternity leave for a minimum of 14 weeks, prenatal leave, exemption from night shifts and protection from risk factors in the workplace [71]. Third, improving prenatal care is one way to reduce the incidence of preterm birth. Compared with the group without prenatal care, receiving prenatal care can reduce the incidence of preterm birth [68]. European countries, such as France, have focused on the primary prevention of pregnancy risks in prenatal care and provided social and financial support for low-risk pregnant women, which has achieved certain results [71,72].

Policymakers in low, low-middle, and middle SDI regions can learn from the experience of countries with similar levels of SDI to their own. Among low, low-middle, and middle SDI countries having a decreasing trend of the burden, some of China’s practices are worth learning from. Over the past 30 years, the ASMR in PM_2.5_-related preterm birth in China decreased from 0.31 to 0.21, by 33.23% (not shown in results). Such burden remained at a fairly low level, which was at the same level as that in high SDI regions. Improving air quality, encouraging hospital-based birth strategy, and improving neonatal care were China’s three ways to reduce the burden of PM_2.5_-related preterm birth. First, in the last several years, the Chinese government has taken many measures to improve air quality. For example, it promulgated the air pollution prevention and control action plan, resulting in the improved air quality [73]. Since 1981, the annual average concentration of air particulate matter in China has continued to decline [74]. In contrast, in South Asia, the burden of PM_2.5_ highlights the huge impact of the lack of policy actions [36]. The implementation of policies to control the use of tobacco and lead is an example worthy of reference. Regulations play an important role in controlling exposure to PM_2.5_, and it does not need action in individual level [75,76].Thus, it is very necessary to promulgate policies to prevent and control air pollution in countries with large and increasing exposure to ambient particulate matter pollution. Second, since 2000, hospital-based birth strategy was encouraged in China in place of community midwifery [77,78,79,80]. As a positive consequence, neonatal death rate decreased by 62% from 1996 to 2008. The neonatal mortality rate for hospital births was much lower than that for home births. Many neonatal deaths were prevented by hospital birth, the proportion of which ranged from 48% to 70% [81]. Third, a cross-sectional study suggested that China had made great progress in improving neonatal care and treatment, along with improving the treatment level of hospitals, increasing the number of neonatal beds and intensive care units, and developing treatment technology [82]. It is demonstrated that the neonatal intensive care is a useful response for the smallest and most preterm of birth infants [83]. Although the cost of neonatal intensive care is expensive, this measure is more economical than other healthcare programs, including hospital-based and intensive programs, and some non-intensive programs [84].

We also noticed that boys bore higher burdens of PM_2.5_-related preterm birth than girls. PM_2.5_ leads to premature birth through complex effects. PM_2.5_ could affect transplacental oxygen and nutrient transport and eventually leads to preterm birth through some complex mechanisms, such as oxidative stress, pulmonary and placental inflammation, blood coagulation, endothelial function, and hemodynamic responses [23]. These mechanisms may be interrelated because oxidative or nitrosative stress may produce adverse effects and lead to the dysfunction of the placenta vascular. Preterm birth can also cause a series of adverse outcomes related to the neural development, including cerebral palsy, cognitive impairment, weak motor coordination, and learning difficulties in the long run [4]. In the above process, male fetuses are more vulnerable than female fetuses. First, male fetuses need more oxygen than female fetuses because they grow faster and hematologic changes may be affected by air pollution [85,86]. One possible reason was that increased blood viscosity more easily affects male fetuses [87,88]. Second, PM_2.5_ exposure was associated with the neural development of fetus [89]. It had a greater adverse effect on male fetuses than on female fetuses. Third, female newborns had more survival advantages in biology [90]. Male newborns were at higher risk of developing adverse outcomes if they were at adverse conditions, for example, preterm birth and older maternal age [91,92,93]. One explanation is that “endogenous” factors play an significant part, for example, genetic factors linked to the pseudo-dominant effect of X-linked genes [94]. It was reported that girls need less healthcare than boys, particularly in South Asia [95,96].

There are some limitations in this study. First of all, PM_2.5_ is a particulate mixed pollutant. Different countries with different economic and social levels have different composition and main effect components of PM_2.5_. Second, due to the lack of other important risk factors, such as occupation, ethnicity, etc., the secondary analysis of GBD data cannot adjust the bias of PM_2.5_-induced preterm birth. Third, the level of exposure to PM_2.5_ is based on the average level of the outdoor environment. It did not deal with indoor pollution because of the lack of the data. Fourth, there is the potential of an ecological fallacy because we studied people at the group level, and PM_2.5_ levels at individual levels may not be closely related to premature births.

## 5. Conclusions

The overall burden of PM_2.5_-related PTB in 2019 was relatively high, mainly concentrated on early neonates, boys, and on low-middle SDI regions. It showed an increasing trend in low-middle and low SDI regions. The association between the burden and the SDI presented an inverted U-shape. It is very necessary to promulgate policies to prevent and control air pollution in countries with large and increasing exposure to PM_2.5_ pollution because it does not need action at an individual level. Focusing on public educational interventions, public and professional policies, and improving the accessibility of prenatal care are other feasible ways for low and low-middle SDI countries. Policy makers should also appropriately allocate medical resources to boys and early newborns.

## Figures and Tables

**Table 1:** Age-standardized mortality rate in PM_2.5_-related preterm birth: rate, percentage of changes, annual percentage changes, and average annual percentage changes in the global and different regions in 1990–2019.

Regions	Age-Standardized Mortality Rate in 1990	Age-Standardized Mortality Rate in 2019	Percentage of Changes in 1990–2019	Annual Percentage Change (Part 1)	Annual Percentage Change (Part 2)	Annual Percentage Change (Part 3)	Average Annual Percentage Change in 1990–2019
Global and Regions divided by Socio-Demographic Index							
Global	0.71 (0.43, 1.11)	0.76 (0.55, 1.02)	7.04%	0.4 (0.3, 0.5) *	2.2 (1.5, 2.9) *	−3.0 (−3.6, −2.3) *	0.2 (0.0, 0.4) *
Low SDI	0.44 (0.14, 1.02)	0.67 (0.36, 1.12)	52.27%	1.5 (1.2, 1.7) *	5.7 (3.2, 8.3) *	−1.5 (−3.2, 0.2)	1.7 (1.1, 2.2) *
Low-middle SDI	0.71 (0.28, 1.42)	1.19 (0.81, 1.65)	67.61%	1.7 (1.5, 1.9) *	4.5 (3.8, 5.3) *	−1.6 (−2.8, −0.4) *	1.9 (1.6, 2.2) *
Middle SDI	0.93 (0.62, 1.28)	0.79 (0.61, 0.98)	−15.05%	1.2 (0.7, 1.7) *	−0.2 (−0.4, 0.0) *	−5.0 (−5.9, −4.0) *	−0.7 (−0.9, −0.4) *
High-middle SDI	0.77 (0.55, 1.04)	0.37 (0.29, 0.46)	−51.95%	−0.2 (−0.9, 0.4)	−2.5 (−2.7, −2.3) *	−6.2 (−7.3, −5.1) *	−2.6 (−2.9, −2.3) *
High SDI	0.35 (0.29, 0.42)	0.13 (0.11, 0.16)	−62.86%	−1.7 (−1.9, −1.4) *	−5.0 (−6.0, −3.9) *	−4.4 (−4.8, −3.9) *	−3.2 (−3.5, −2.9) *
Regions divided by Geography							
Central Europe	0.70 (0.45, 0.93)	0.17 (0.11, 0.23)	−75.71%	−1.6 (−4.1, 1.0)	−7.9 (−10.3, −5.5) *	−4.3 (−4.5, −4.1) *	−4.6 (−5.1, −4.1) *
Australasia	0.20 (0.02, 0.57)	0.06 (0.01, 0.16)	−70.00%	−8.3 (−9.8, −6.8) *	−1.2 (−1.9, −0.6) *	−6.7 (−9.8, −3.6) *	−4.2 (−4.9, −3.5) *
Central Asia	0.47 (0.25, 0.76)	0.52 (0.31, 0.78)	10.64%	2.2 (0.7, 3.7) *	0.6 (0.5, 0.7) *	−3.8 (−5.9, −1.5) *	0.3 (0.0, 0.7) *
Central Latin America	0.73 (0.42, 1.09)	0.32 (0.21, 0.45)	−56.16%	0.5 (−0.4, 1.3)	−4.9 (−6.9, −3.0) *	−3.5 (−3.9, −3.1) *	−2.6 (−3.1, −2.1) *
Tropical Latin America	0.59 (0.30, 1.03)	0.29 (0.18, 0.45)	−50.85%	−0.5 (−0.9, 0.0) *	−2.2 (−2.8, −1.6) *	−4.7 (−5.2, −4.3) *	−2.5 (−2.8, −2.2) *
Caribbean	0.37 (0.18, 0.66)	0.41 (0.19, 0.74)	10.81%	0.6 (0.4, 0.8) *	4.4 (−2.4, 11.6)	−1.8 (−2.9, −0.7) *	0.5 (−0.2, 1.2) *
Eastern Europe	0.38 (0.27, 0.53)	0.08 (0.05, 0.12)	−78.95%	−5.0 (−5.4, −4.5) *	−14.4 (−30.7, 5.7)	0.6 (−18.5, 24.2)	−5.6 (−7.9, −3.2) *
Southeast Asia	0.59 (0.28, 1.05)	0.40 (0.25, 0.59)	−32.20%	1.4 (0.4, 2.3) *	−1.0 (−1.1, −0.9) *	−3.3 (−3.6, −3.1) *	−1.4 (−1.6, −1.2) *
Western Europe	0.29 (0.22, 0.38)	0.09 (0.06, 0.12)	−68.97%	−4.6 (−5.0, −4.1) *	−3.8 (−4.1, −3.6) *	−1.9 (−8.2, 4.9)	−4.0 (−4.4, −3.5) *
Southern Latin America	0.58 (0.14, 1.29)	0.28 (0.06, 0.64)	−51.72%	−3.2 (−3.8, −2.7) *	−0.6 (−1.2, −0.1) *	−4.1 (−4.8, −3.3) *	−2.5 (−2.8, −2.2) *
High-income Asia Pacific	0.15 (0.08, 0.26)	0.04 (0.02, 0.07)	−73.33%	−6.5 (−8.4, −4.5) *	0.6 (−19.7, 26.1)	−4.5 (−5.3, −3.8) *	−4.6 (−6.8, −2.4) *
Andean Latin America	0.91 (0.39, 1.61)	0.44 (0.21, 0.74)	−51.65%	−2.8 (−3.1, −2.6) *	−1.3 (−1.7, −1.0) *	−3.9 (−4.4, −3.4) *	−2.5 (−2.7, −2.4) *
Oceania	0.13 (0.03, 0.36)	0.17 (0.04, 0.44)	30.77%	0.6 (0.4, 0.8) *	3.5 (−2.2, 9.5)	0.4 (−0.1, 0.9)	0.8 (0.3, 1.4) *
East Asia	0.48 (0.22, 0.83)	0.31 (0.23, 0.39)	−35.42%	5.1 (0.8, 9.6) *	−1.4 (−1.8, −1.0) *	−7.0 (−9.7, −4.2) *	−1.5 (−2.3, −0.8) *
North Africa and Middle East	1.86 (1.33, 2.49)	0.95 (0.70, 1.28)	−48.92%	−1.0 (−1.2, −0.8) *	−1.9 (−2.1, −1.8) *	−5.1 (−5.5, −4.6) *	−2.3 (−2.4, −2.1) *
South Asia	0.94 (0.37, 1.84)	1.49 (1.05, 2.02)	58.51%	1.6 (1.3, 1.9) *	4.9 (3.9, 5.9) *	−2.8 (−4.3, −1.2) *	1.7 (1.3, 2.1) *
Central Sub-Saharan Africa	0.32 (0.09, 0.80)	0.53 (0.24, 0.98)	65.62%	0.2 (0.0, 0.3) *	6.2 (5.0, 7.3) *	1.7 (0.6, 2.8) *	1.6 (1.3, 1.9) *
Eastern Sub-Saharan Africa	0.17 (0.05, 0.43)	0.26 (0.12, 0.48)	52.94%	−1.3 (−3.9, 1.4)	2.4 (2.2, 2.6) *	−1.0 (−3.5, 1.7)	1.4 (0.9, 1.9) *
High-income North America	0.38 (0.28, 0.49)	0.16 (0.11, 0.21)	−57.89%	−3.6 (−4.2, −3.0) *	−1.8 (−2.4, −1.1) *	−3.3 (−3.5, −3.1) *	−3.0 (−3.2, −2.7) *
Southern Sub-Saharan Africa	0.84 (0.51, 1.26)	1.22 (0.80, 1.78)	45.24%	1.7 (0.8, 2.7) *	4.8 (4.4, 5.2) *	−2.2 (−2.6, −1.8) *	1.5 (1.2, 1.7) *
Western Sub-Saharan Africa	0.53 (0.20, 1.21)	1.13 (0.63, 1.84)	113.21%	2.5 (2.3, 2.6) *	6.9 (4.4, 9.4) *	0.2 (−0.9, 1.2)	2.7 (2.3, 3.0) *

The annual percentage change in each part represents the change trend between two year points. The numbers in brackets represent the 95% confidence interval. * *p*-value < 0.05.

**Table 2:** Age-standardized disability-adjusted life years rate in PM_2.5_-related preterm birth: rate, percentage of changes, annual percentage changes, and average annual percentage changes in the global and different regions in 1990–2019.

Regions	Age-Standardized Disability-Adjusted Life Years Rate in 1990	Age-Standardized Disability-Adjusted Life Years Rate in 2019	Percentage of Changes in 1990–2019	Annual Percentage Change (Part 1)	Annual Percentage Change (Part 2)	Annual Percentage Change (Part 3)	Average Annual Percentage Change in 1990–2019
Global and Regions divided by Socio-Demographic Index							
Global	63.21 (37.82, 98.71)	67.71 (48.59, 91.17)	7.12%	0.4 (0.3, 0.5) *	2.2 (1.4, 2.9) *	−2.9 (−3.6, −2.2) *	0.2 (0.0, 0.4) *
Low SDI	39.43 (12.68, 91.07)	59.69 (32.08, 99.72)	51.38%	1.4 (1.2, 1.6) *	5.8 (3.2, 8.4) *	−1.4 (−3.1, 0.4)	1.7 (1.1, 2.2) *
Low-middle SDI	63.28 (25.08, 125.96)	105.99 (71.96, 147.00)	67.49%	1.7 (1.5, 1.9) *	4.5 (3.7, 5.3) *	−1.6 (−2.8, −0.3) *	1.9 (1.6, 2.2) *
Middle SDI	82.58 (55.38, 113.47)	69.89 (54.12, 87.25)	−15.37%	1.2 (0.7, 1.6) *	−0.2 (−0.4, 0.0) *	−5.0 (−5.9, −4.0) *	−0.7 (−0.9, −0.5) *
High middle SDI	68.27 (48.56, 92.15)	32.84 (25.47, 41.02)	−51.90%	0.7 (−0.4, 1.9)	−2.4 (−2.5, −2.2) *	−6.5 (−7.5, −5.4) *	−2.6 (−2.8, −2.3) *
High-SDI	31.05 (25.42, 37.63)	11.75 (9.39, 14.57)	−62.16%	−1.7 (−1.8, −1.6) *	−5.0 (−5.6, −4.4) *	−4.4 (−4.6, −4.1) *	−3.2 (−3.4, −3.1) *
Regions divided by Geography							
Central Europe	62.23 (40.00, 82.66)	14.75 (9.70, 20.60)	−76.30%	−1.5 (−4.0, 1.1)	−7.9 (−10.2, −5.5) *	−4.4 (−4.6, −4.2) *	−4.6 (−5.1, −4.1) *
Australasia	17.77 (1.70, 51.06)	5.47 (0.57, 14.10)	−69.22%	−7.5 (−8.2, −6.7) *	−1.2 (−1.6, −0.8) *	−6.7 (−8.4, −4.8) *	−4.1 (−4.5, −3.7) *
Central Asia	42.15 (22.44, 67.35)	46.35 (27.97, 69.60)	9.96%	1.7 (0.7, 2.8) *	0.6 (0.4, 0.7) *	−3.7 (−5.9, −1.5) *	0.3 (0.0, 0.6) *
Central Latin America	64.97 (37.19, 96.88)	28.10 (18.56, 40.46)	−56.75%	0.5 (−0.4, 1.4)	−5.0 (−7.0, −2.9) *	−3.5 (−3.9, −3.1) *	−2.6 (−3.1, −2.1) *
Tropical Latin America	52.52 (26.44, 91.57)	26.14 (15.81, 39.64)	−50.23%	−0.5 (−0.9, 0.0) *	−2.2 (−2.8, −1.6) *	−4.6 (−5.1, −4.2) *	−2.5 (−2.7, −2.2) *
Caribbean	33.25 (16.04, 58.34)	36.44 (16.97, 65.50)	9.59%	0.6 (0.4, 0.7) *	4.8 (−2.1, 12.1)	−1.9 (−3.0, −0.8) *	0.5 (−0.2, 1.2) *
Eastern Europe	33.86 (23.64, 47.31)	6.93 (4.43, 10.26)	−79.53%	0.0 (−9.4, 10.4)	−5.3 (−5.9, −4.7) *	−9.3 (−12.3, −6.2) *	−5.6 (−6.8, −4.5) *
Southeast Asia	52.10 (24.99, 93.34)	35.73 (22.47, 52.13)	−31.42%	1.5 (0.7, 2.3) *	−1.0 (−1.1, −0.9) *	−3.3 (−3.5, −3.0) *	−1.4 (−1.5, −1.2) *
Western Europe	25.87 (19.23, 34.00)	7.67 (5.10, 10.92)	−70.35%	−4.6 (−4.8, −4.4) *	−3.7 (−3.8, −3.6) *	−4.6 (−5.9, −3.4) *	−4.1 (−4.2, −4.0) *
Southern Latin America	51.27 (12.10, 114.70)	25.10 (5.40, 56.80)	−51.04%	−3.2 (−3.7, −2.7) *	−0.6 (−1.1, −0.1) *	−4.1 (−4.7, −3.4) *	−2.5 (−2.8, −2.2) *
High-income Asia Pacific	13.75 (6.71, 23.11)	3.74 (1.87, 6.32)	−72.80%	−7.3 (−8.9, −5.6) *	0.0 (−14.8, 17.4)	−4.1 (−4.6, −3.6) *	−4.6 (−6.2, −3.0) *
Andean Latin America	81.13 (35.10, 143.43)	38.98 (18.97, 65.41)	−51.95%	−3.0 (−3.3, −2.7) *	−1.4 (−1.7, −1.1) *	−3.9 (−4.4, −3.4) *	−2.5 (−2.7, −2.4) *
Oceania	11.25 (2.61, 32.32)	14.88 (3.55, 39.15)	32.27%	0.7 (0.6, 0.9) *	3.0 (−1.3, 7.5)	0.7 (0.4, 1.1) *	1.0 (0.5, 1.4) *
East Asia	42.23 (19.54, 73.90)	27.45 (20.51, 34.5)	−35.00%	5.4 (1.1, 9.8) *	−1.4 (−1.8, −1.1) *	−7.1 (−9.8, −4.3) *	−1.5 (−2.3, −0.8) *
North Africa and Middle East	165.27 (117.83, 221.37)	84.93 (61.99, 113.69)	−48.61%	−1.0 (−1.2, −0.8) *	−1.9 (−2.1, −1.8) *	−5.0 (−5.4, −4.6) *	−2.3 (−2.4, −2.1) *
South Asia	83.40 (33.15, 163.94)	132.13 (93.40, 179.52)	58.43%	1.7 (1.5, 2.0) *	5.3 (4.0, 6.6) *	−2.9 (−4.5, −1.4) *	1.8 (1.3, 2.2) *
Central Sub-Saharan Africa	28.80 (7.78, 71.11)	47.16 (21.27, 87.17)	63.75%	0.3 (0.2, 0.4) *	7.4 (6.2, 8.6) *	1.4 (0.6, 2.2) *	1.7 (1.5, 1.9) *
Eastern Sub-Saharan Africa	15.25 (4.71, 37.98)	22.91 (10.39, 42.77)	50.23%	−0.9 (−3.5, 1.8)	2.4 (2.1, 2.6) *	−1.1 (−3.7, 1.6)	1.4 (0.9, 2.0) *
High-income North America	33.77 (24.89, 43.13)	13.89 (9.78, 18.98)	−58.87%	−3.7 (−4.5, −2.9) *	−2.0 (−2.5, −1.4) *	−3.2 (−3.4, −3.0) *	−2.9 (−3.2, −2.7) *
Southern Sub-Saharan Africa	74.31 (45.02, 111.60)	108.68 (71.13, 158.01)	46.25%	1.5 (0.6, 2.4) *	5.2 (4.8, 5.6) *	−1.8 (−2.1, −1.5) *	1.5 (1.2, 1.8) *
Western Sub-Saharan Africa	47.56 (17.66, 107.89)	100.06 (56.43, 163.93)	110.39%	2.4 (2.3, 2.6) *	7.0 (4.6, 9.4) *	0.2 (−0.8, 1.2)	2.7 (2.3, 3.0) *

The annual percentage change in each part represents the change trend between two year points. The numbers in brackets represent the 95% confidence interval. * *p*-value < 0.05.

## Data Availability

Data are available in a publicly accessible repository. Publicly available datasets were analyzed in this study. These data can be found by using the Global Health Data Exchange (GHDx, http://ghdx.healthdata.org/gbd-results-tool (accessed on 4 January 2022)).

## Supplementary Materials

The following supporting information can be downloaded at: https://www.mdpi.com/article/10.3390/ijerph191610068/s1.
