# Supplementary material for: The Association between the Burden of PM2.5-Related Neonatal Preterm Birth and Socio-Demographic Index from 1990 to 2019: A Global Burden Study
Source: Int J Environ Res Public Health. 2022 Aug 15;19(16):10068. doi: 10.3390/ijerph191610068 (PMC9408320; doi:10.3390/ijerph191610068)
Supplement: Supplementary file 1 [file ijerph-19-10068-s001.zip › ijerph-1758595-supplementary.pdf]

Supplementary Material

The association between the burden of $PM_{2.5}$-related neonatal preterm birth and Socio-demographic index from 1990 to 2019: a global burden study

Zeyu Tang[1], Jinzhu Jia[1,2*]

[1]Department of Biostatistics, School of Public Health, Peking University, No.38, Xueyuan Road, Beijing 100871, China

[2]Center for Statistical Science, Peking Universeity, 5 Summer Palace Road, Beijing 100871, China

[*] Correspondence: jzjia@math.pku.edu.cn.

Part 1 (Figures S1–S21): Trends in the age-standardized mortality rate of $PM_{2.5}$-related preterm birth in 21 regions from 1990 to 2019.

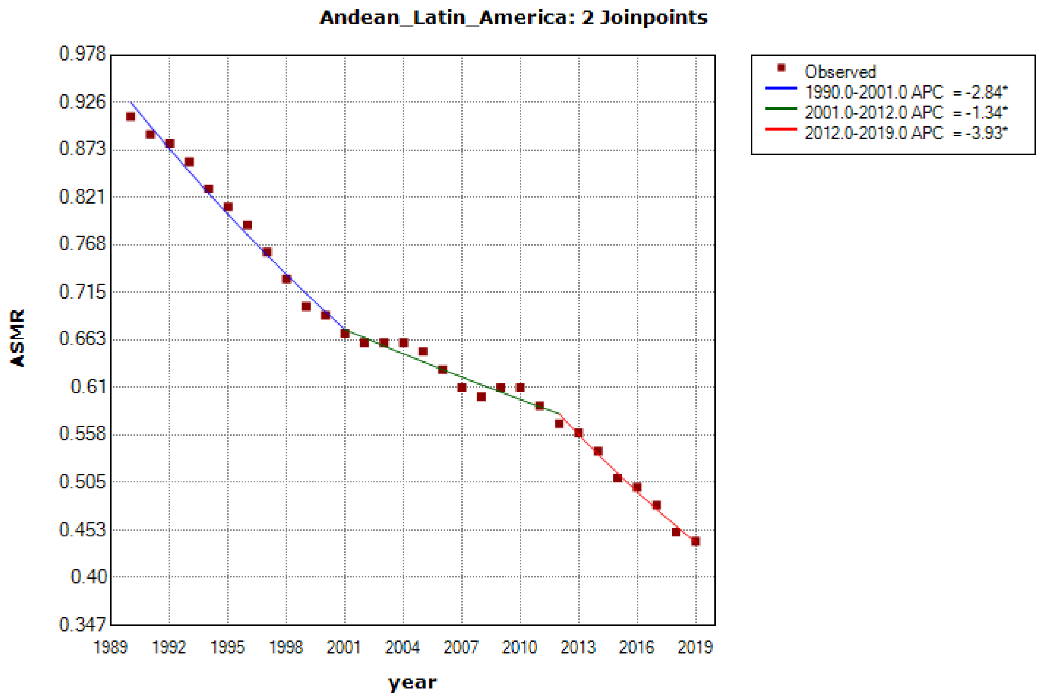

Figure S1 Trends in the age-standardized mortality rate (ASMR, per 100,000 population) of $PM_{2.5}$-related preterm birth in Andean Latin America from 1990 to 2019. $^{*}P$<0.05

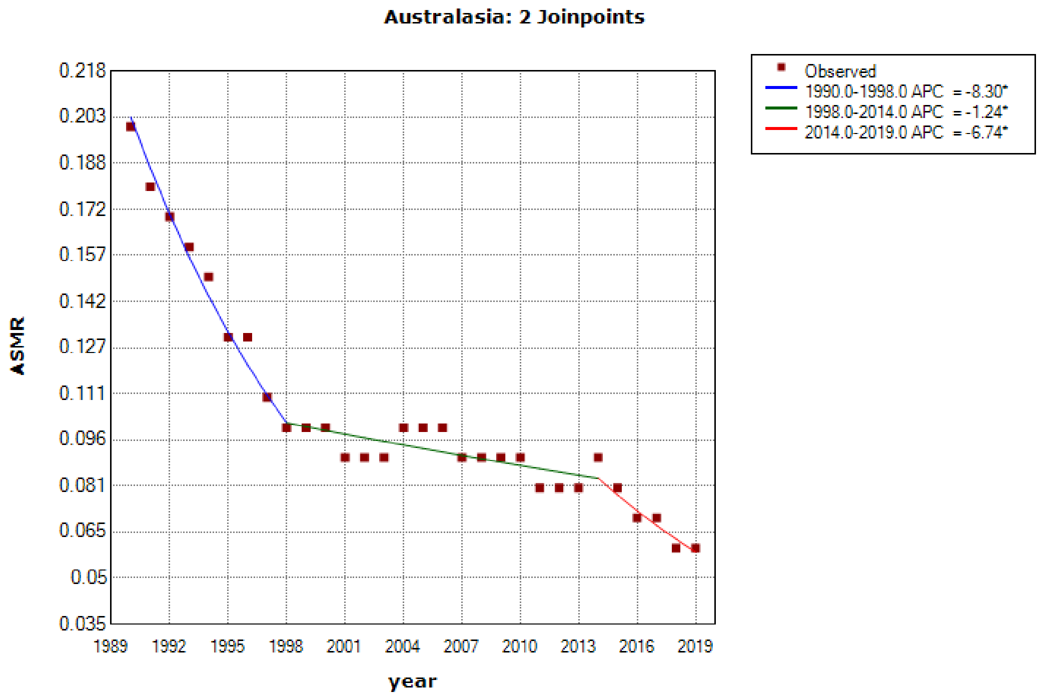

Figure S2 Trends in the age-standardized mortality rate (ASMR, per 100,000 population) of $PM_{2.5}$-related preterm birth in Australasia from 1990 to 2019. $^{*}P$<0.05

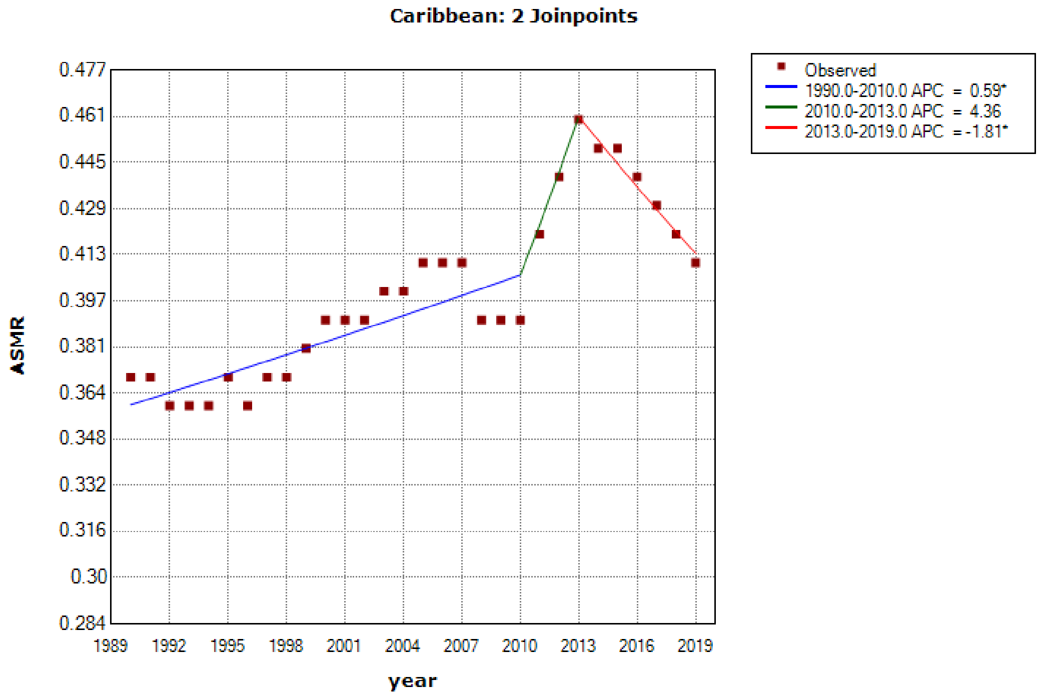

Figure S3 Trends in the age-standardized mortality rate (ASMR, per 100,000 population) of $PM_{2.5}$-related preterm birth in Caribbean from 1990 to 2019. $^{*}P$<0.05

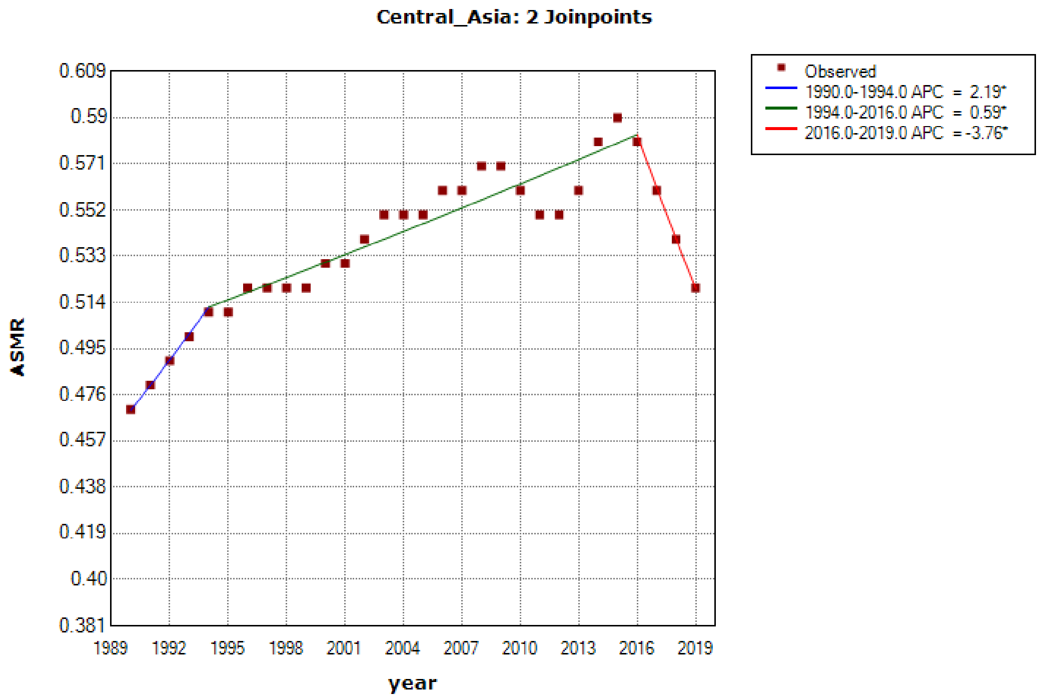

Figure S4 Trends in the age-standardized mortality rate (ASMR, per 100,000 population) of $PM_{2 \cdot 5}$-related preterm birth in Central Asia from 1990 to 2019. $^{*}P$<0.05

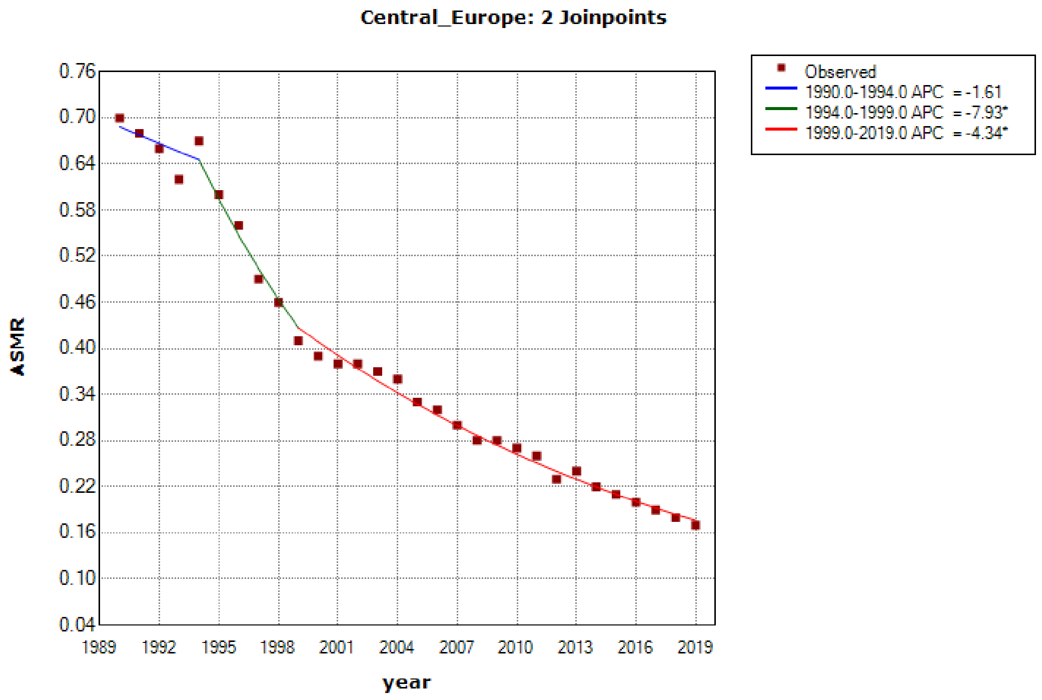

Figure S5 Trends in the age-standardized mortality rate (ASMR, per 100,000 population) of $PM_{2 \cdot 5}$-related preterm birth in Central Europe from 1990 to 2019. $^{*}P$<0.05

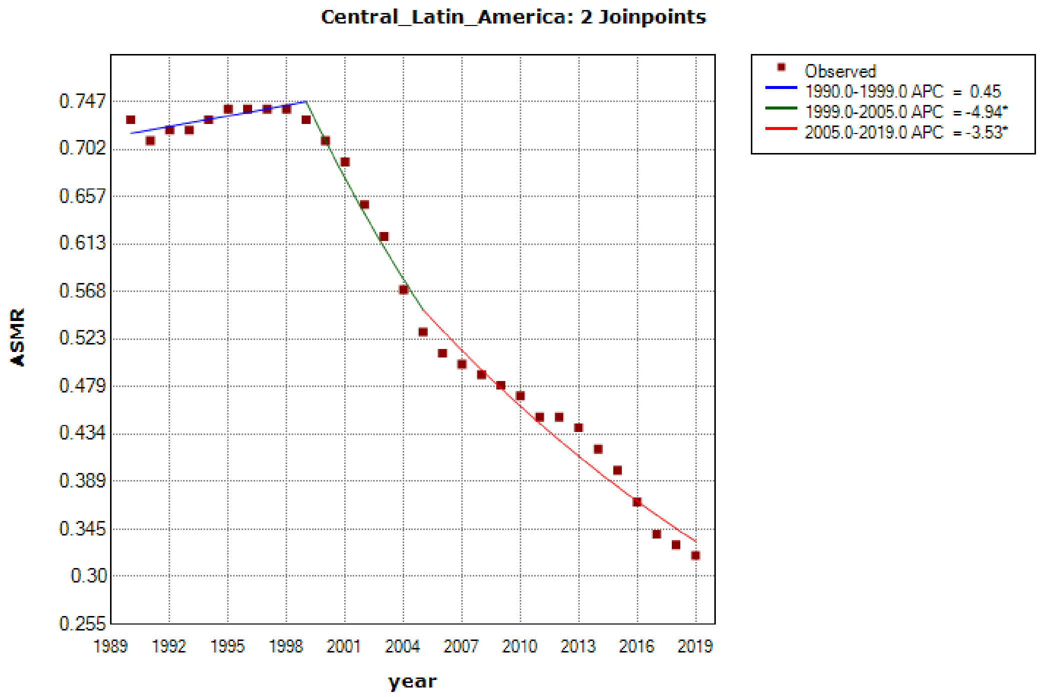

Figure S6 Trends in the age-standardized mortality rate (ASMR, per 100,000 population) of $PM_{2.5}$-related preterm birth in Central Latin America from 1990 to 2019. $^{*}P$<0.05

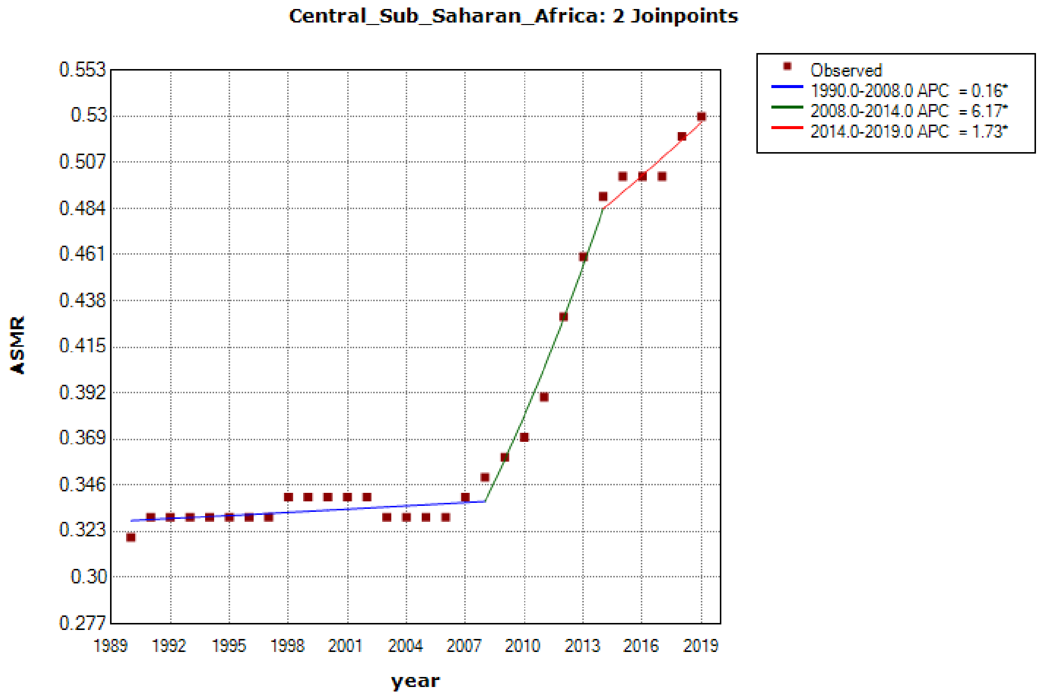

Figure S7 Trends in the age-standardized mortality rate (ASMR, per 100,000 population) of $PM_{2.5}$-related preterm birth in Central Sub Saharan Africa from 1990 to 2019. $^{*}P$<0.05

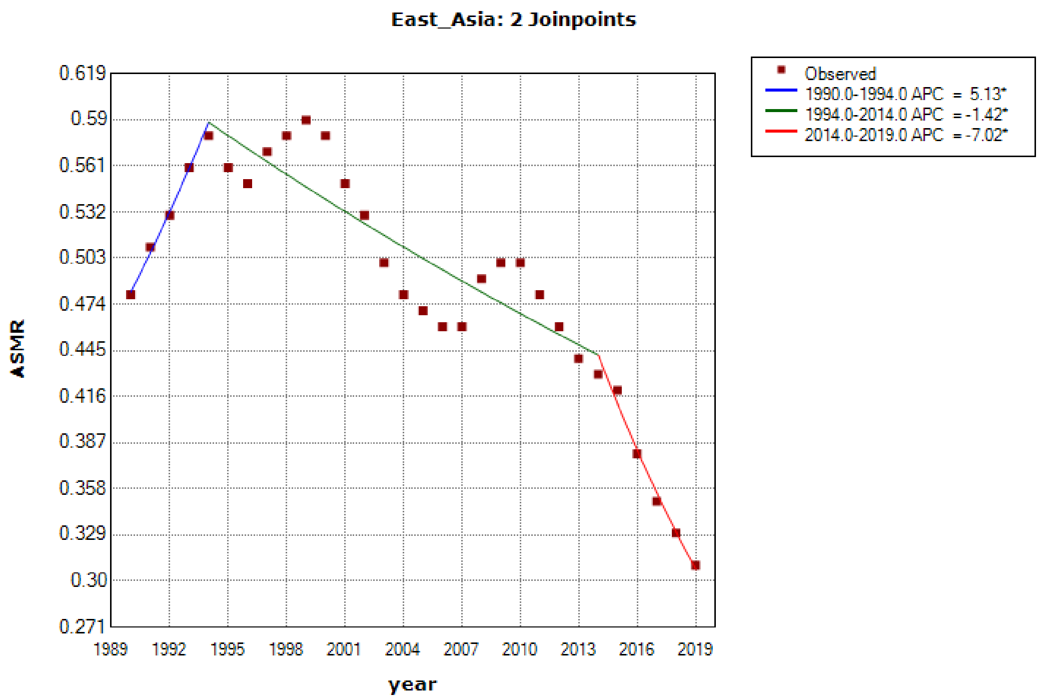

Figure S8 Trends in the age-standardized mortality rate (ASMR, per 100,000 population) of $PM_{2.5}$-related preterm birth in East Asia from 1990 to 2019. $^{*}P$<0.05

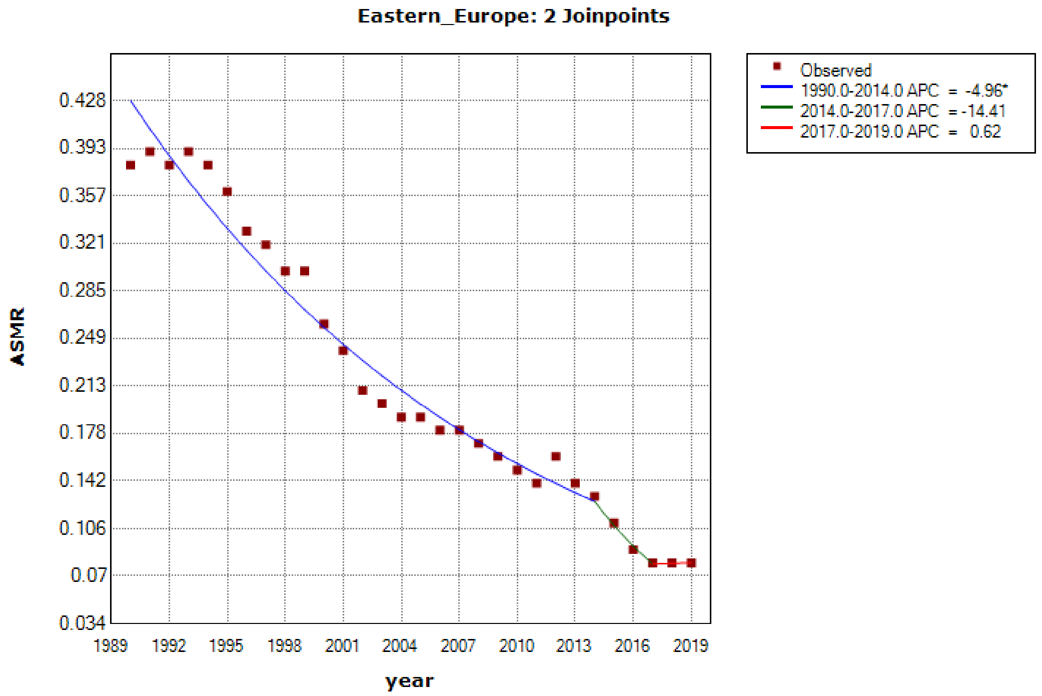

Figure S9 Trends in the age-standardized mortality rate (ASMR, per 100,000 population) of $PM_{2.5}$-related preterm birth in Eastern Europe from 1990 to 2019. $^{*}P$<0.05

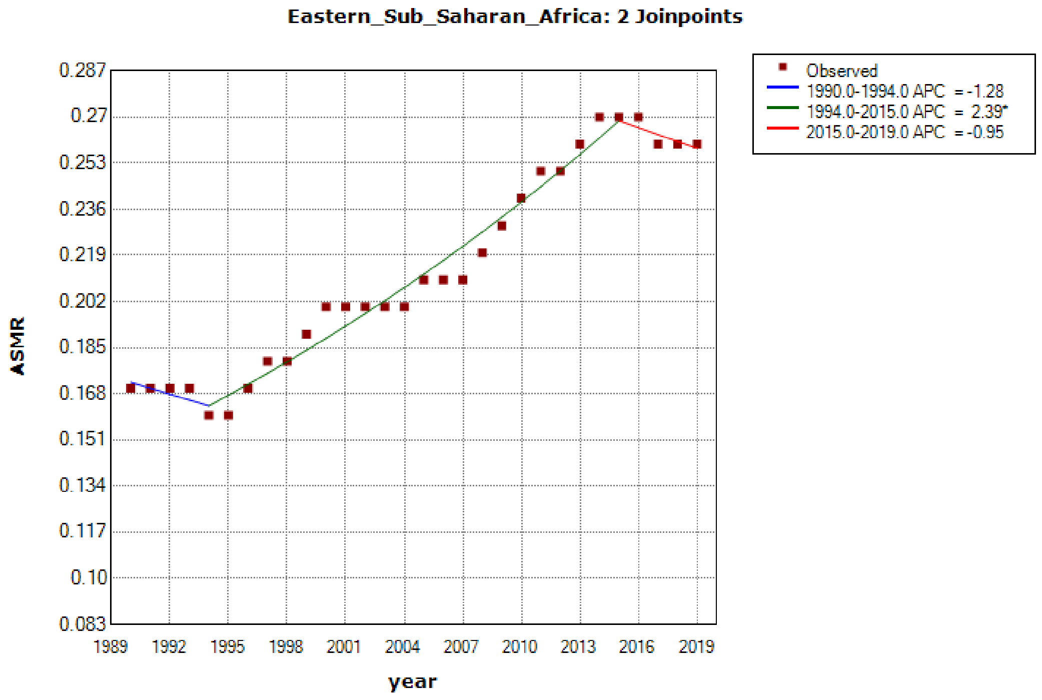

Figure S10 Trends in the age-standardized mortality rate (ASMR, per 100,000 population) of $PM_{2.5}$-related preterm birth in Eastern Sub Saharan Africa from 1990 to 2019. $^{*}P<0.05$

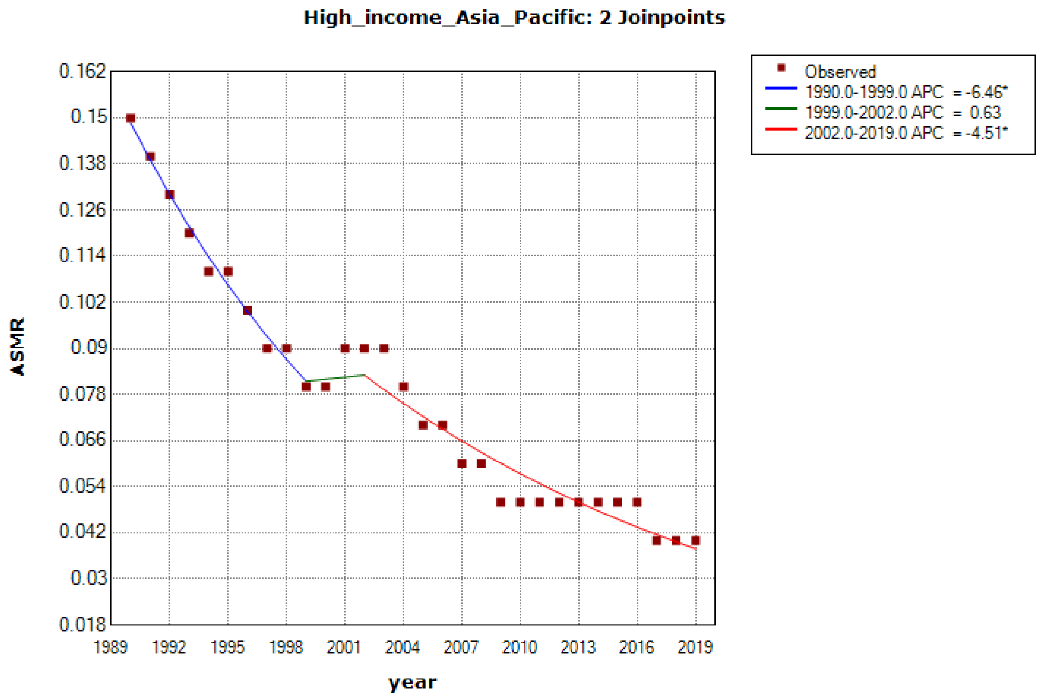

Figure S11 Trends in the age-standardized mortality rate (ASMR, per 100,000 population) of $PM_{2.5}$-related preterm birth in High income Asia Pacific from 1990 to 2019. $^{*}P<0.05$

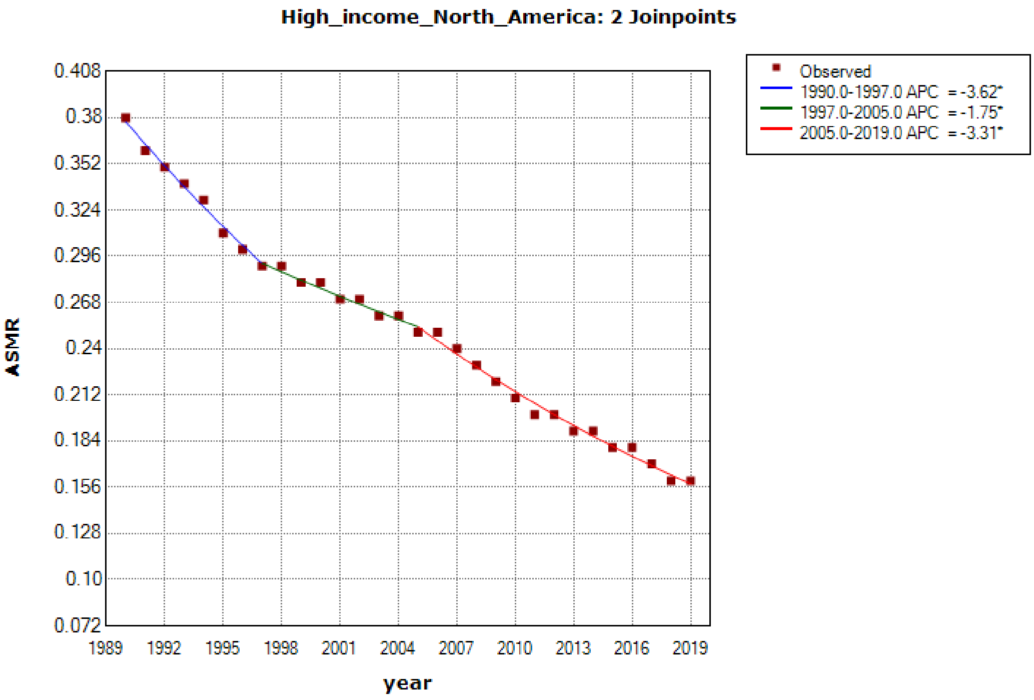

Figure S12 Trends in the age-standardized mortality rate (ASMR, per 100,000 population) of $PM_{2.5}$-related preterm birth in High income North America from 1990 to 2019. $^{*}P<0.05$

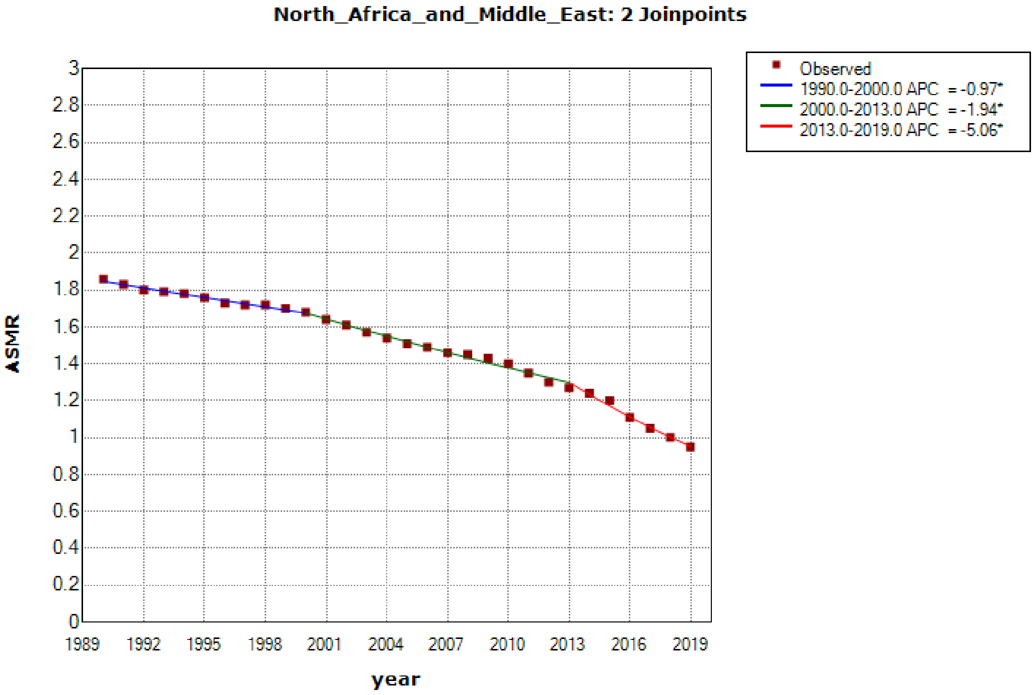

Figure S13 Trends in the age-standardized mortality rate (ASMR, per 100,000 population) of $PM_{2.5}$-related preterm birth in North Africa and Middle East from 1990 to 2019. $^{*}P<0.05$

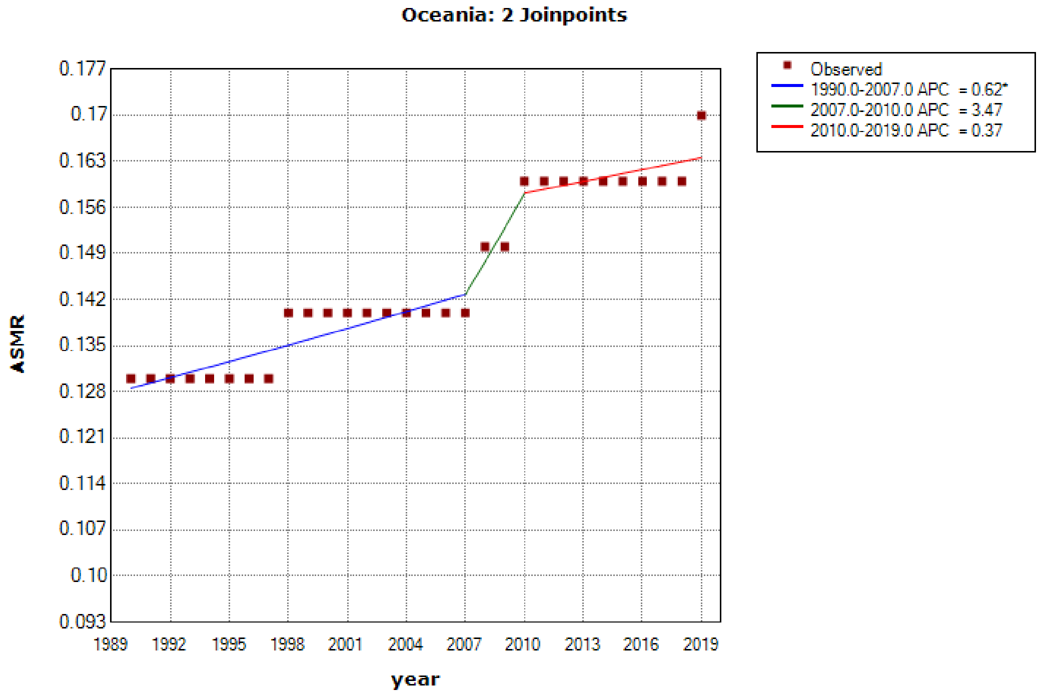

Figure S14 Trends in the age-standardized mortality rate (ASMR, per 100,000 population) of $PM_{2.5}$-related preterm birth in Oceania from 1990 to 2019. $^{*}P<0.05$

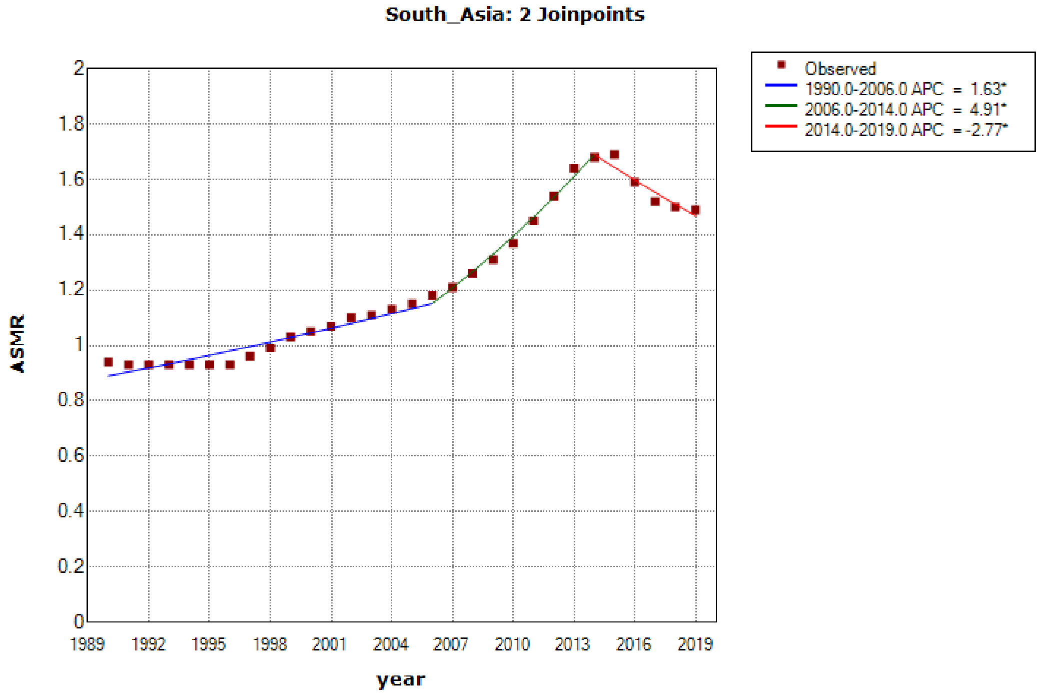

Figure S15 Trends in the age-standardized mortality rate (ASMR, per 100,000 population) of $PM_{2.5}$-related preterm birth in South Asia from 1990 to 2019. $^{*}P<0.05$

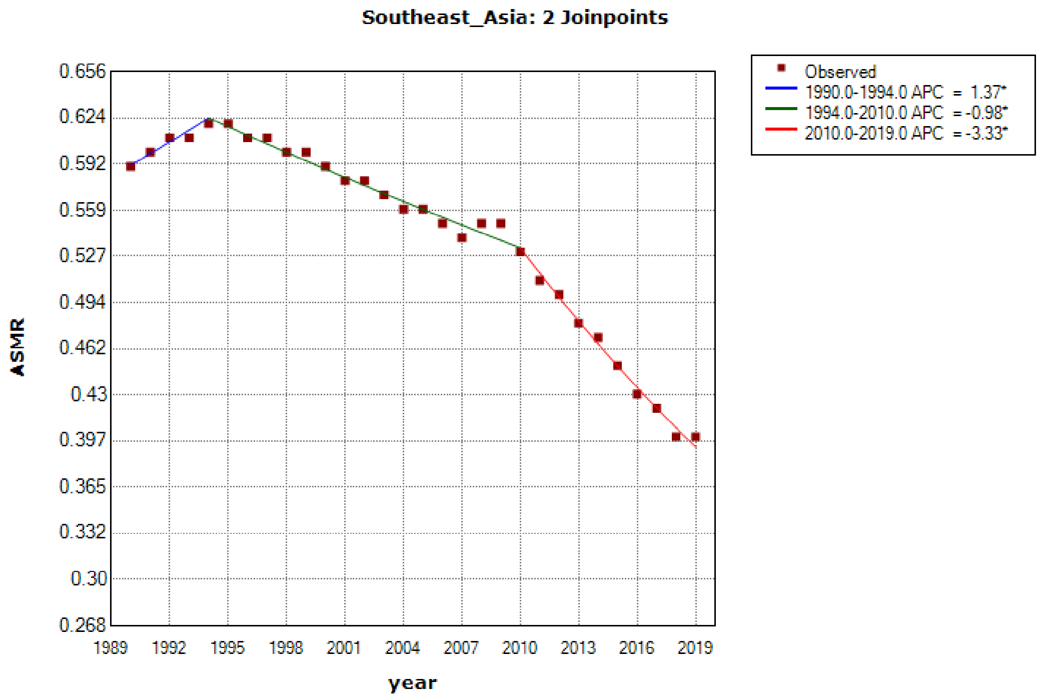

Figure S16 Trends in the age-standardized mortality rate (ASMR, per 100,000 population) of $PM_{2.5}$-related preterm birth in Southeast Asia from 1990 to 2019. $^*P<0.05$

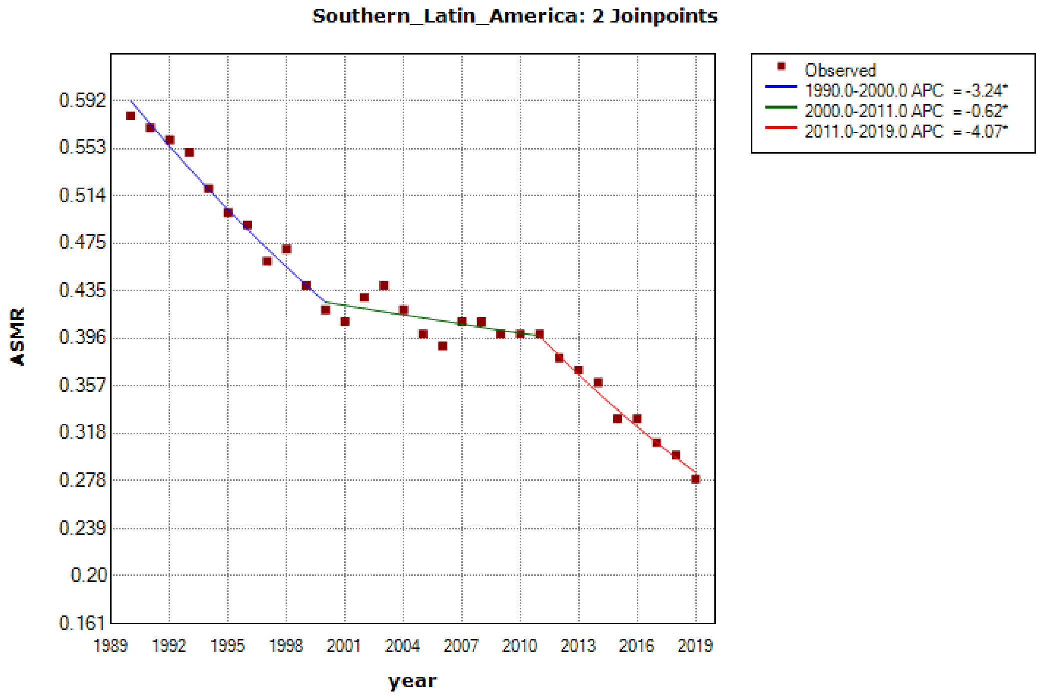

Figure S17 Trends in the age-standardized mortality rate (ASMR, per 100,000 population) of $PM_{2.5}$-related preterm birth in Southern Latin America from 1990 to 2019. $^*P<0.05$

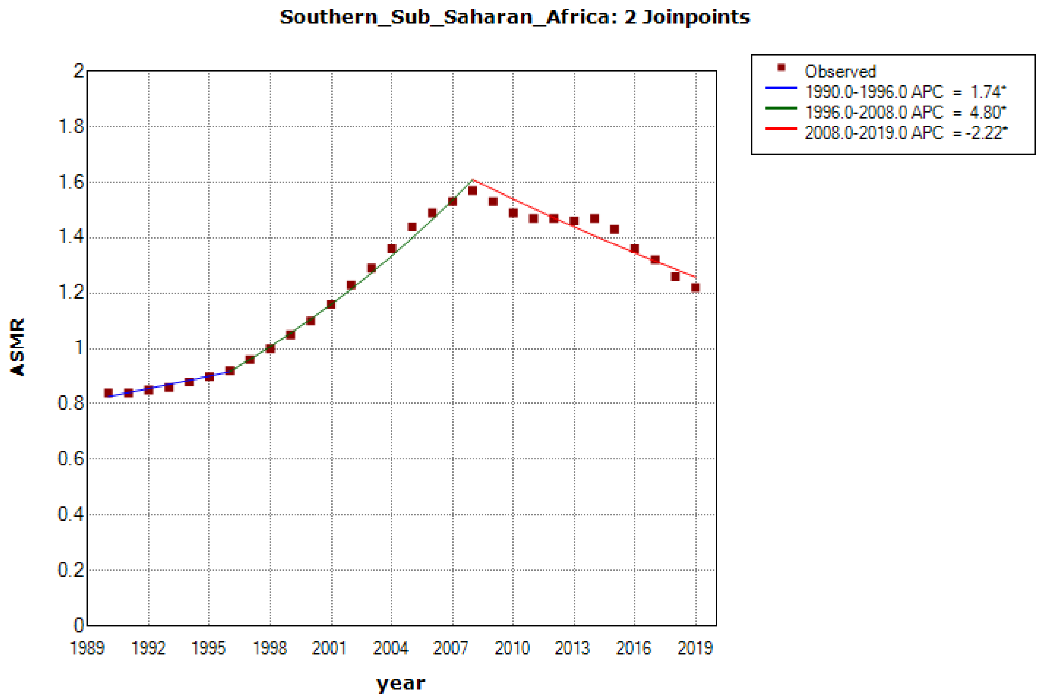

Figure S18 Trends in the age-standardized mortality rate (ASMR, per 100,000 population) of $PM_{2.5}$-related preterm birth in Southern Sub Saharan Africa from 1990 to 2019. $^{*}P<0.05$

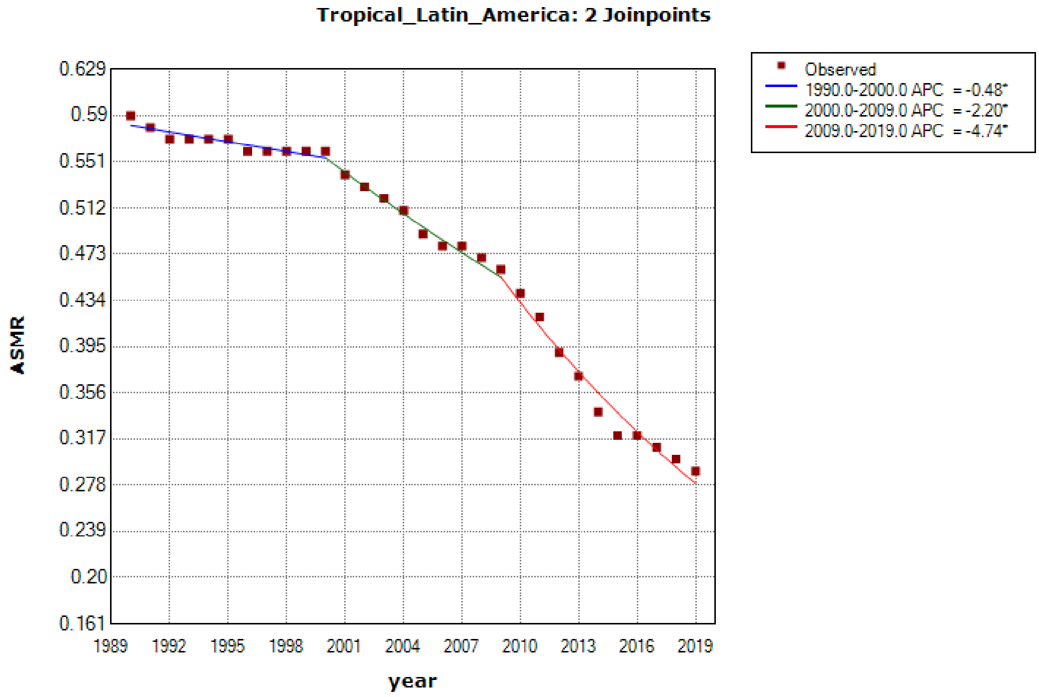

Figure S19 Trends in the age-standardized mortality rate (ASMR, per 100,000 population) of $PM_{2.5}$-related preterm birth in Tropical Latin America from 1990 to 2019. $^{*}P<0.05$

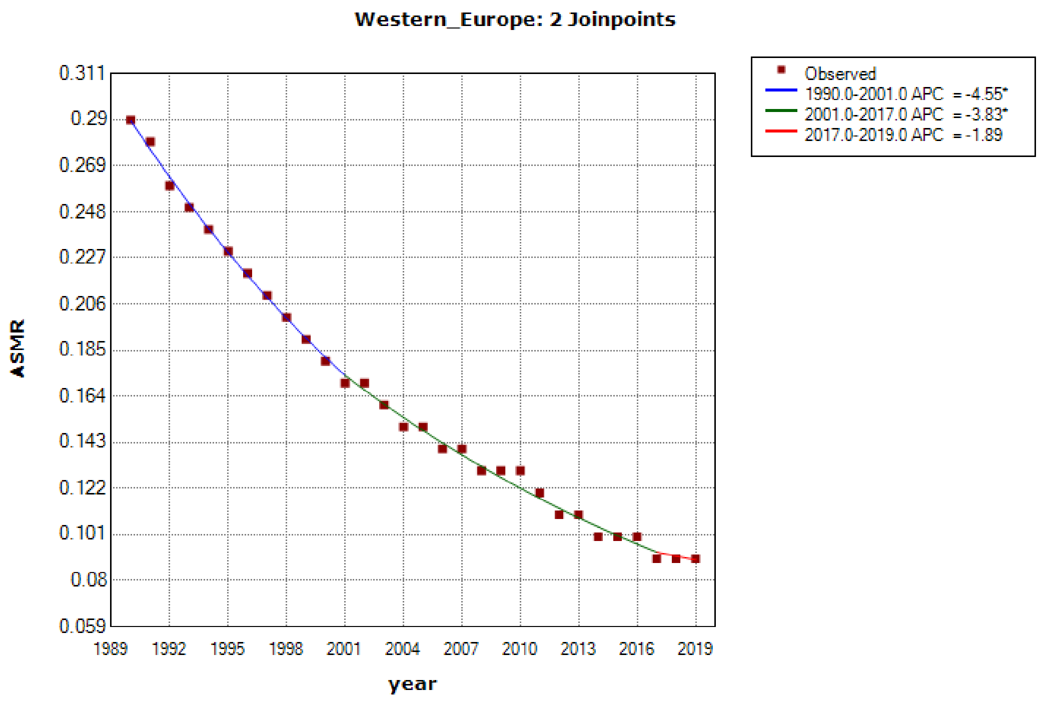

Figure S20 Trends in the age-standardized mortality rate (ASMR, per 100,000 population) of $PM_{2.5}$-related preterm birth in Western Europe from 1990 to 2019. $^{*}P$<0.05

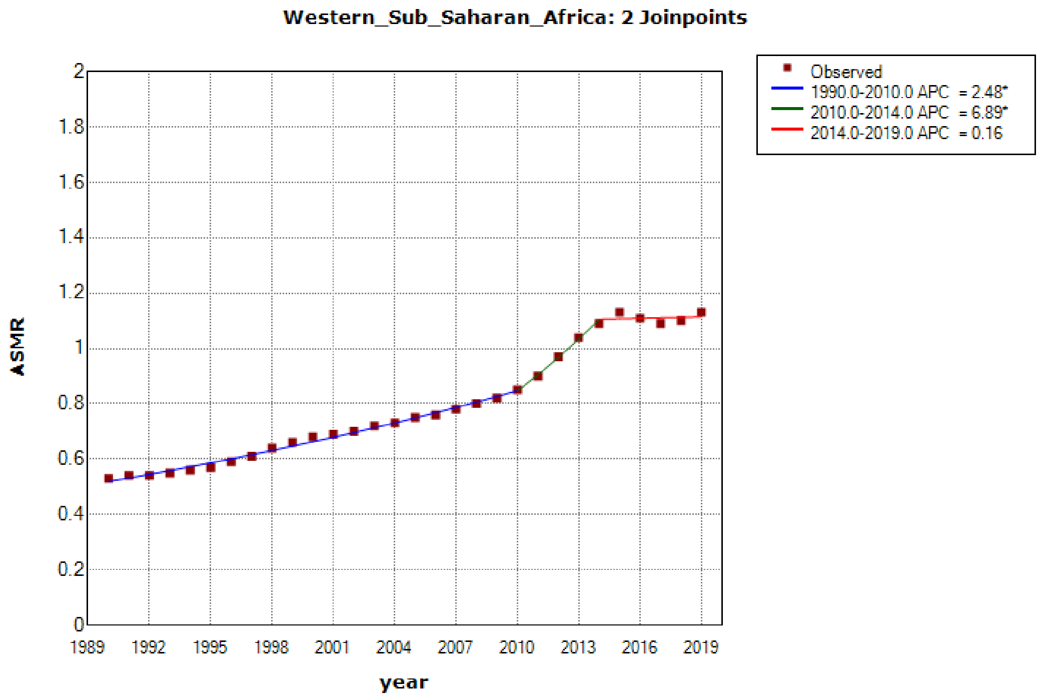

Figure S21 Trends in the age-standardized mortality rate (ASMR, per 100,000 population) of $PM_{2.5}$-related preterm birth in Western Sub Saharan Africa from 1990 to 2019. $^{*}P$<0.05

Part 2 (Fig.S22-S42): Trends in the age-standardized disability-adjusted life rate of $PM_{2.5}$-related preterm birth in 21 regions from 1990 to 2019.

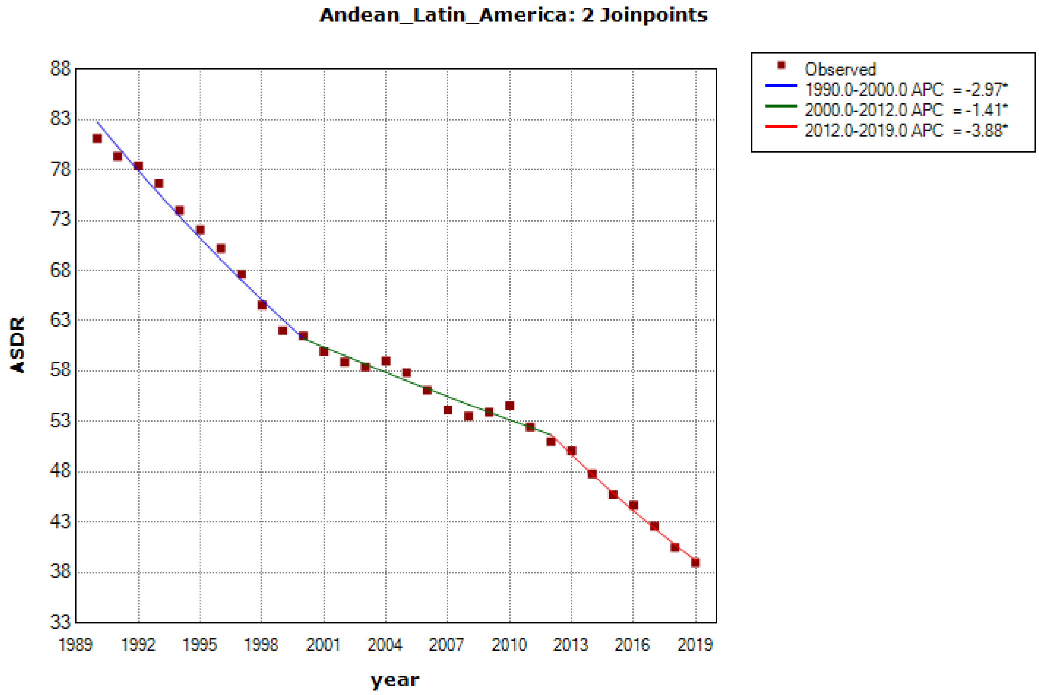

Figure S22 Trends in the age-standardized disability-adjusted life rate (ASDR, per 100,000 population) of $PM_{2.5}$-related preterm birth in Andean Latin America from 1990 to 2019. $^{*}P<0.05$

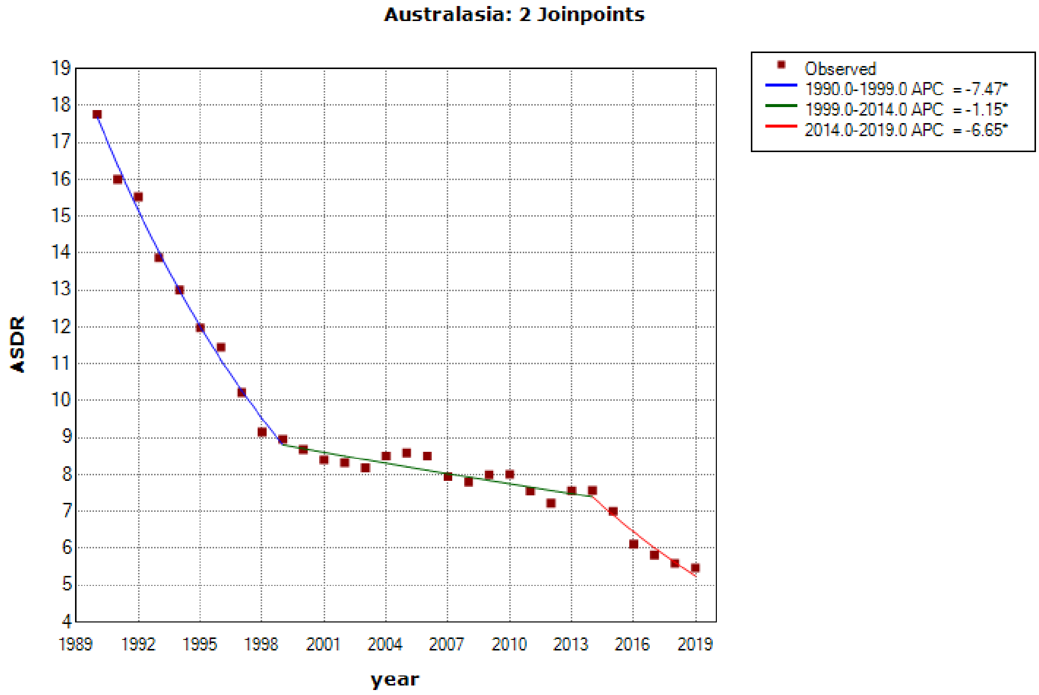

Figure S23 Trends in the age-standardized disability-adjusted life rate (ASDR, per 100,000 population) of $PM_{2.5}$-related preterm birth in Australasia from 1990 to 2019. $^{*}P<0.05$

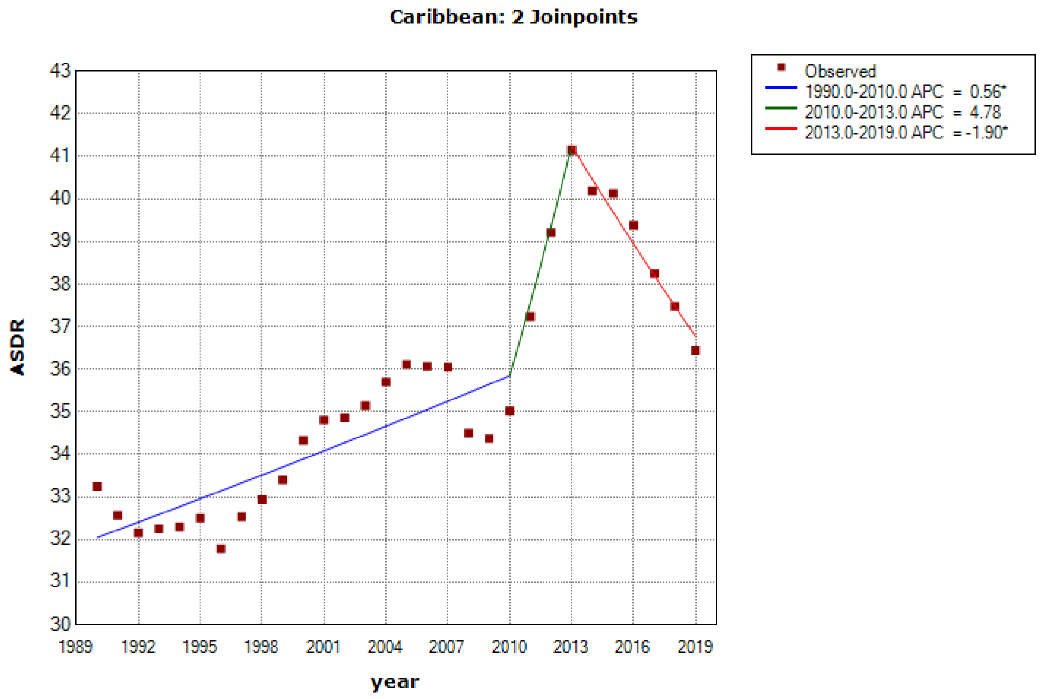

Figure S24 Trends in the age-standardized disability-adjusted life rate (ASDR, per 100,000 population) of $PM_{2.5}$-related preterm birth in Caribbean from 1990 to 2019. $^{*}P$<0.05

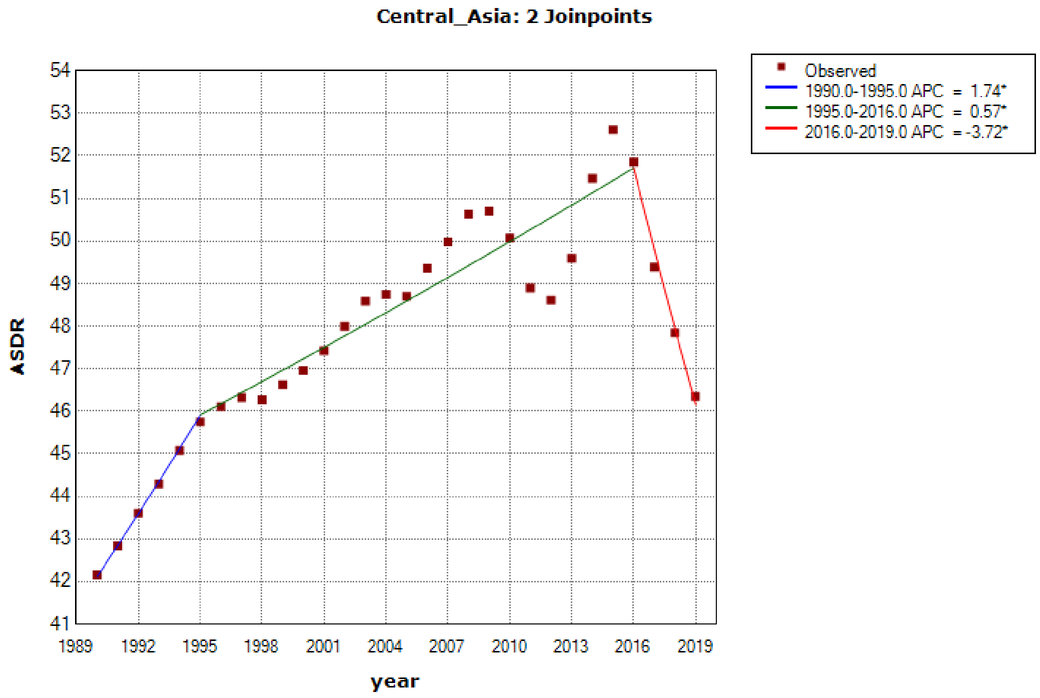

Figure S25 Trends in the age-standardized disability-adjusted life rate (ASDR, per 100,000 population) of $PM_{2.5}$-related preterm birth in Central Asia from 1990 to 2019. $^{*}P$<0.05

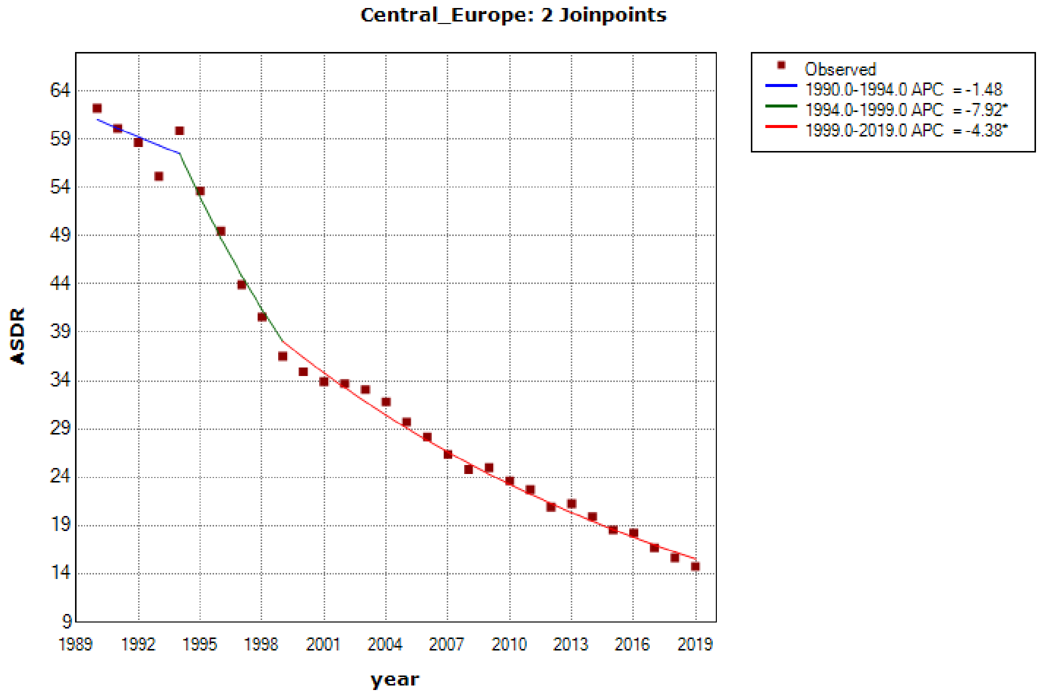

Figure S26 Trends in the age-standardized disability-adjusted life rate (ASDR, per 100,000 population) of $PM_{2.5}$-related preterm birth in Central Europe from 1990 to 2019. $^{*}P<0.05$

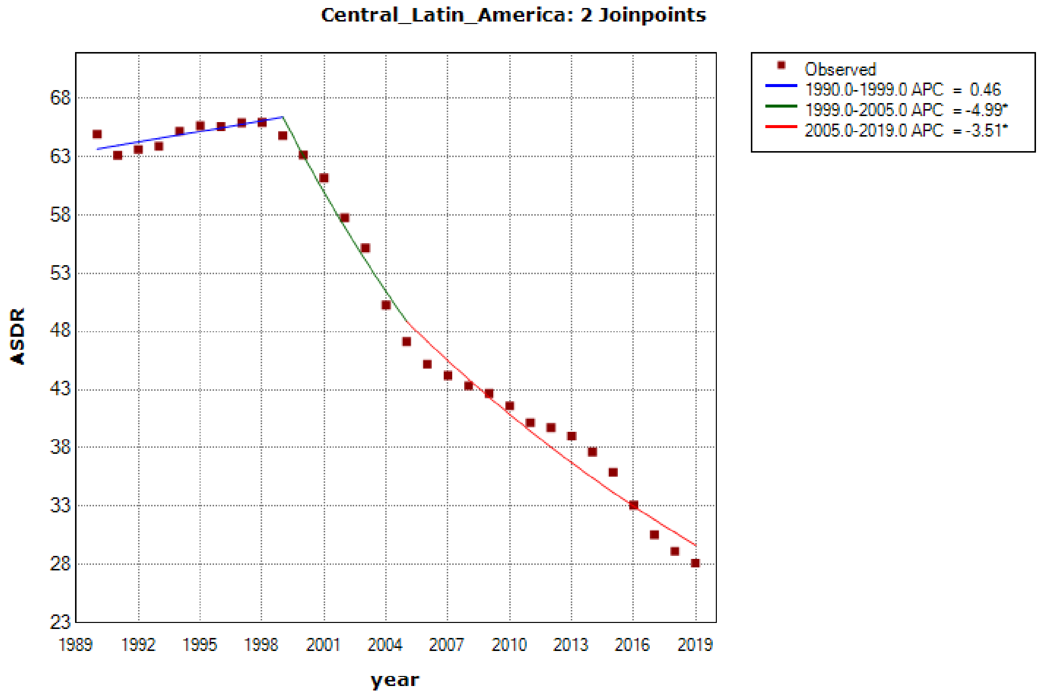

Figure S27 Trends in the age-standardized disability-adjusted life rate (ASDR, per 100,000 population) of $PM_{2.5}$-related preterm birth in Central Latin America from 1990 to 2019. $^{*}P<0.05$

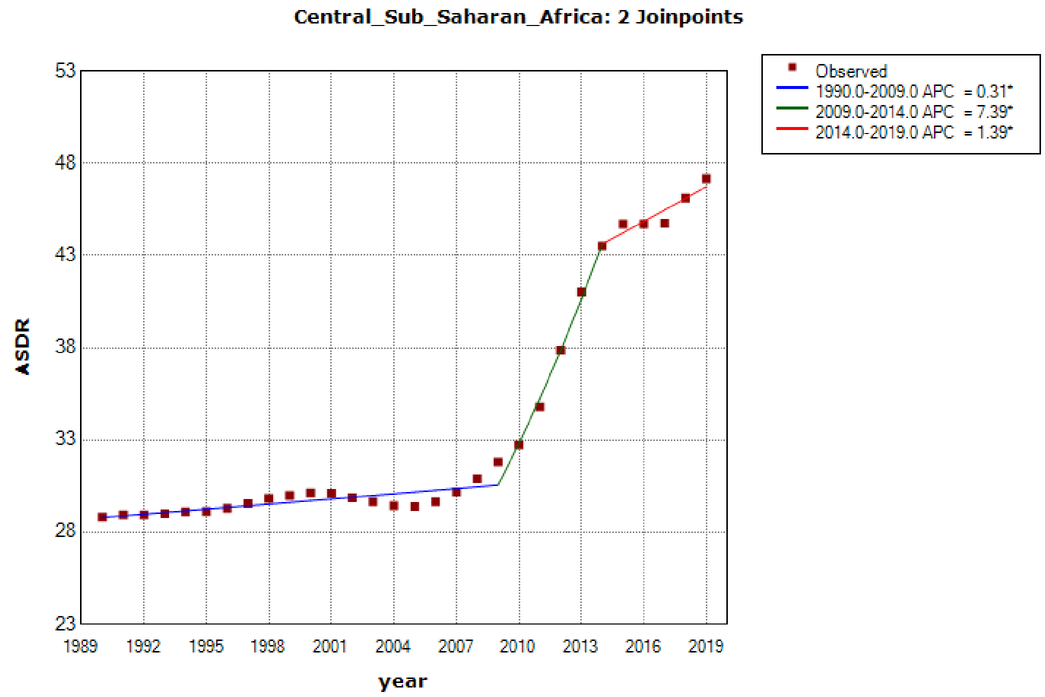

Figure S28 Trends in the age-standardized disability-adjusted life rate (ASDR, per 100,000 population) of $PM_{2\cdot5}$-related preterm birth in Central Sub Saharan Africa from 1990 to 2019. $^{*}P$<0.05

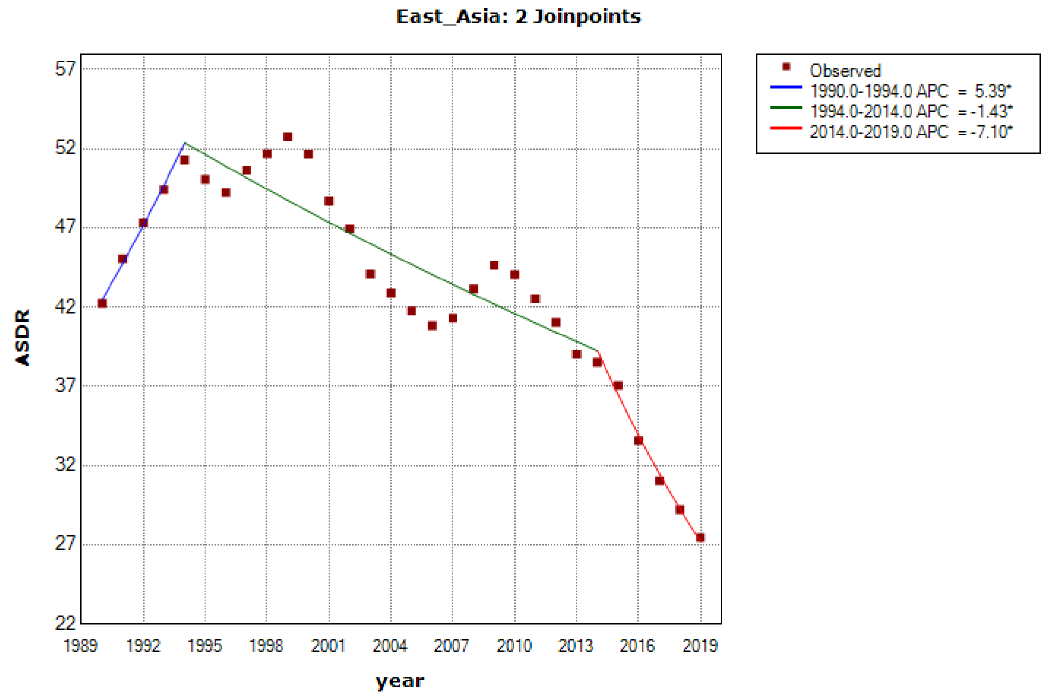

Figure S29 Trends in the age-standardized disability-adjusted life rate (ASDR, per 100,000 population) of $PM_{2\cdot5}$-related preterm birth in East Asia from 1990 to 2019. $^{*}P$<0.05

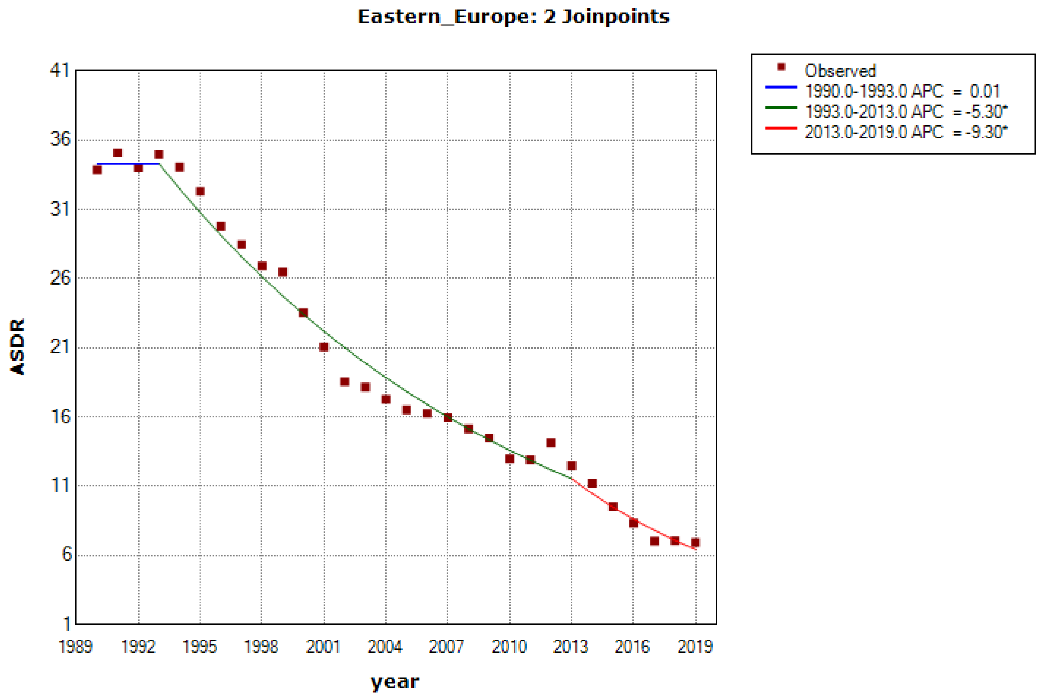

Figure S30 Trends in the age-standardized disability-adjusted life rate (ASDR, per 100,000 population) of $PM_{2.5}$-related preterm birth in Eastern Europe from 1990 to 2019. *P<0.05

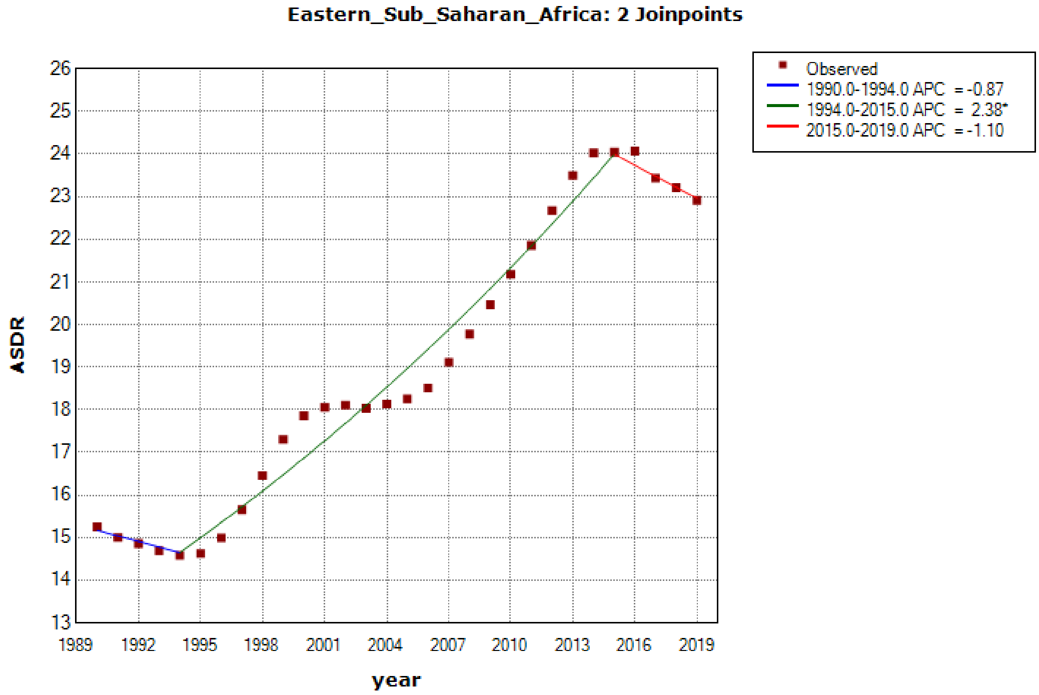

Figure S31 Trends in the age-standardized disability-adjusted life rate (ASDR, per 100,000 population) of $PM_{2.5}$-related preterm birth in Eastern Sub Saharan Africa from 1990 to 2019. *P<0.05

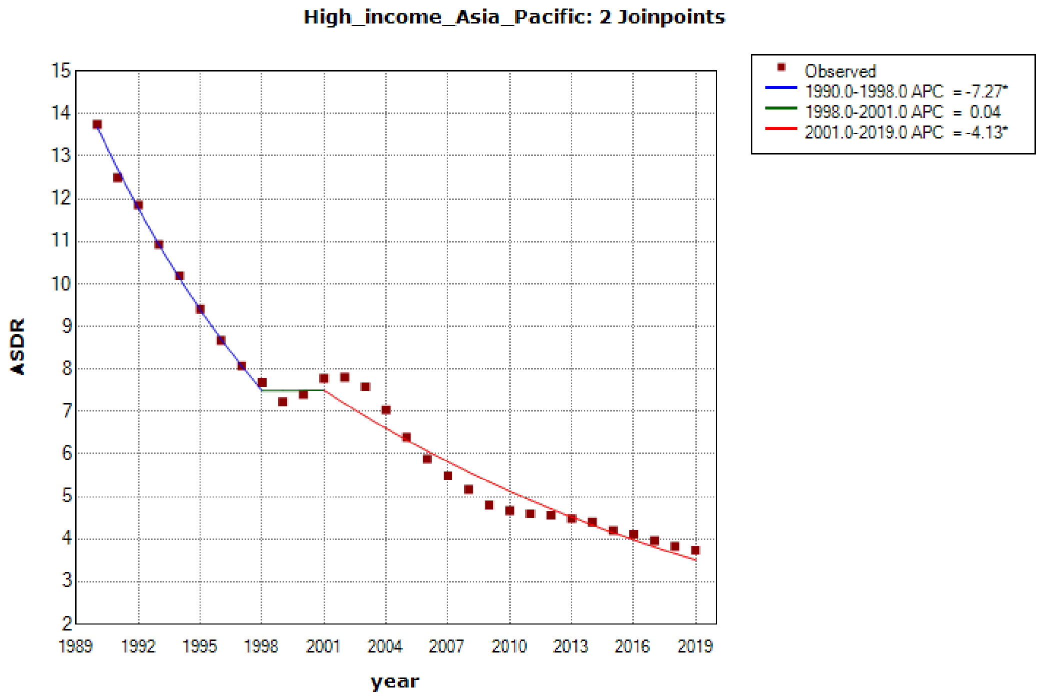

Figure S32 Trends in the age-standardized disability-adjusted life rate (ASDR, per 100,000 population) of $PM_{2.5}$-related preterm birth in High income Asia Pacific from 1990 to 2019. $^{*}P$<0.05

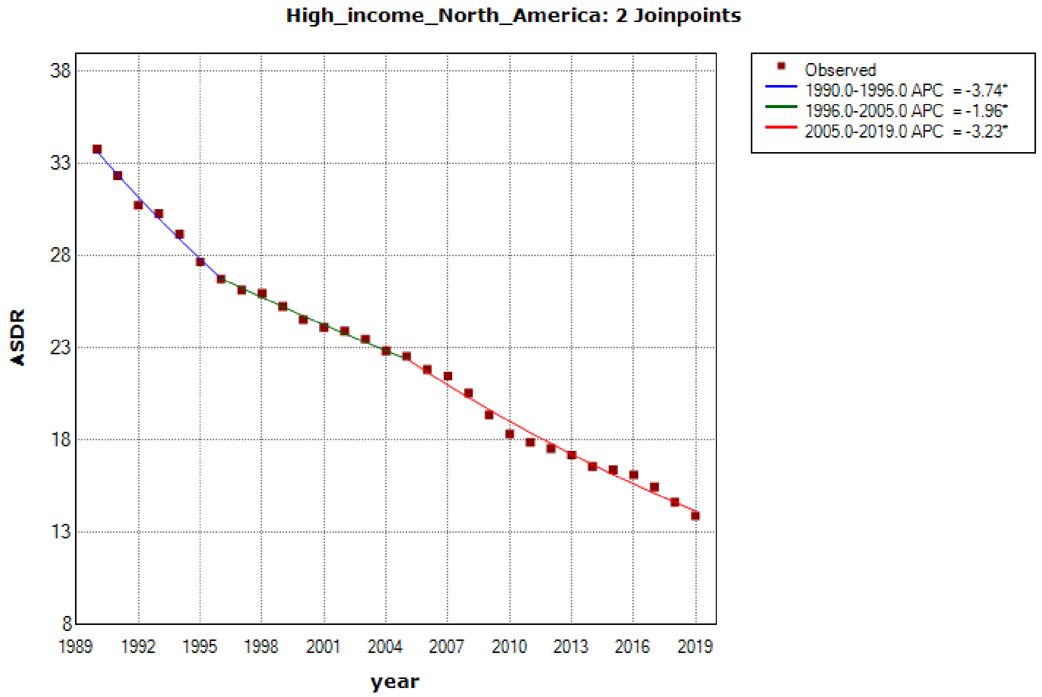

Figure S33 Trends in the age-standardized disability-adjusted life rate (ASDR, per 100,000 population) of $PM_{2.5}$-related preterm birth in High income North America from 1990 to 2019. $^{*}P$<0.05

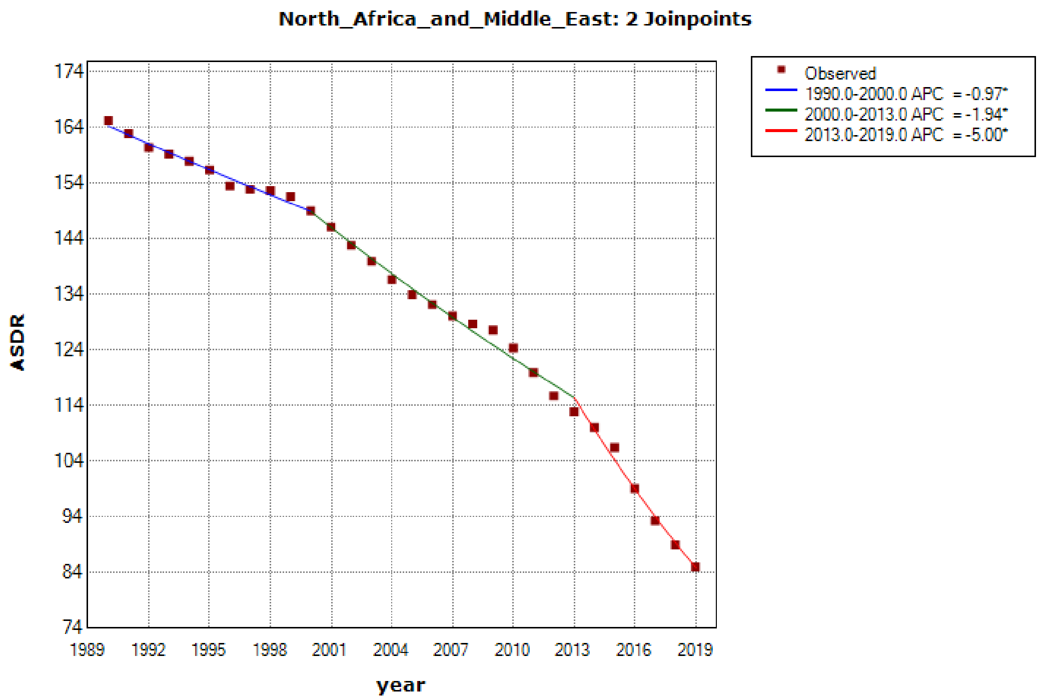

Figure S34 Trends in the age-standardized disability-adjusted life rate (ASDR, per 100,000 population) of $PM_{2.5}$-related preterm birth in North Africa and Middle East from 1990 to 2019. $^{*}P$<0.05

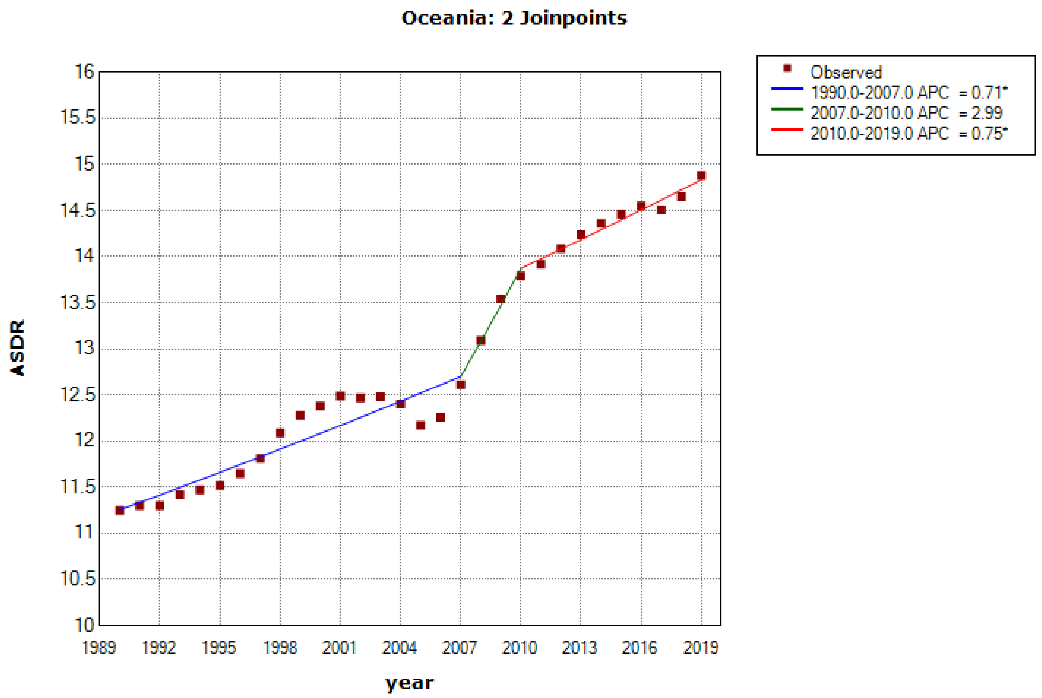

Figure S35 Trends in the age-standardized disability-adjusted life rate (ASDR, per 100,000 population) of $PM_{2.5}$-related preterm birth in Oceania from 1990 to 2019. $^{*}P$<0.05

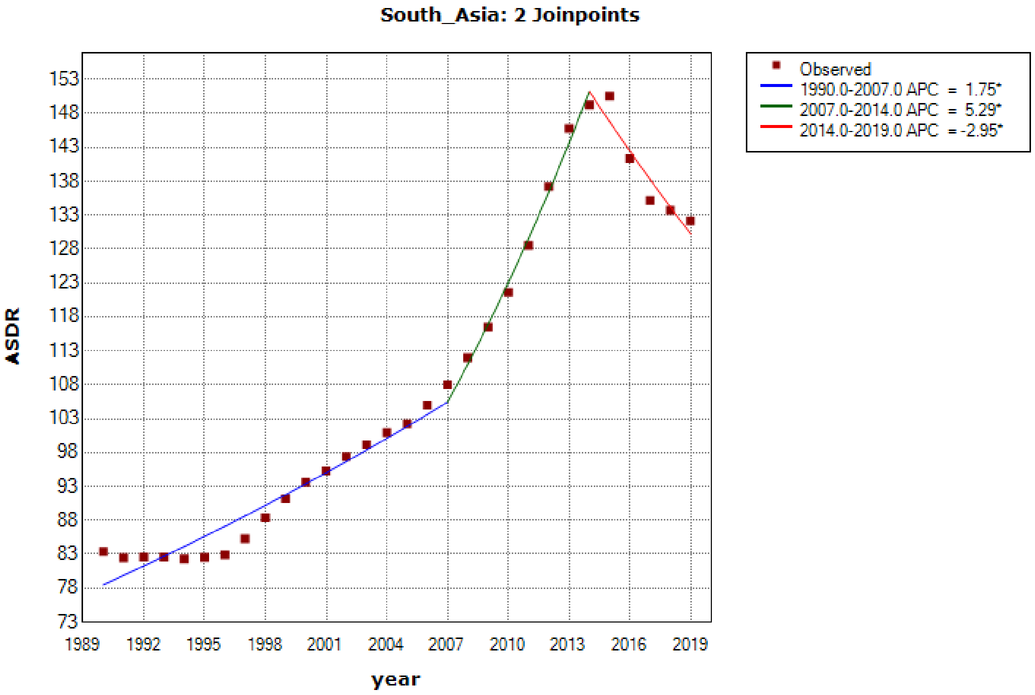

Figure S36 Trends in the age-standardized disability-adjusted life rate (ASDR, per 100,000 population) of $PM_{2.5}$-related preterm birth in South Asia from 1990 to 2019. $^{*}P$<0.05

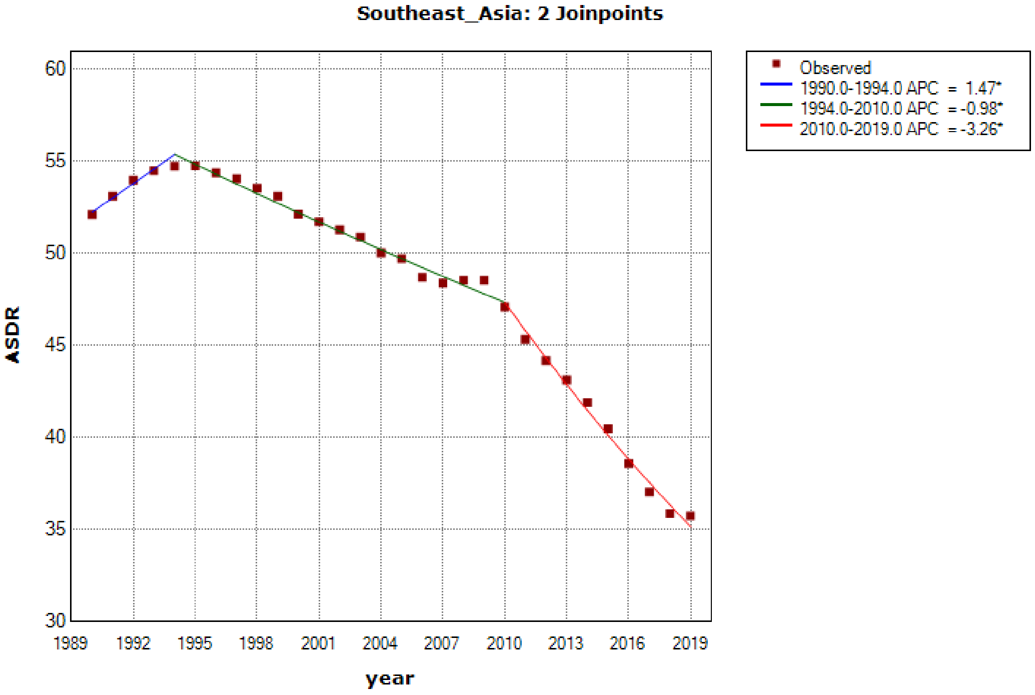

Figure S37 Trends in the age-standardized disability-adjusted life rate (ASDR, per 100,000 population) of $PM_{2.5}$-related preterm birth in Southeast Asia from 1990 to 2019. $^{*}P$<0.05

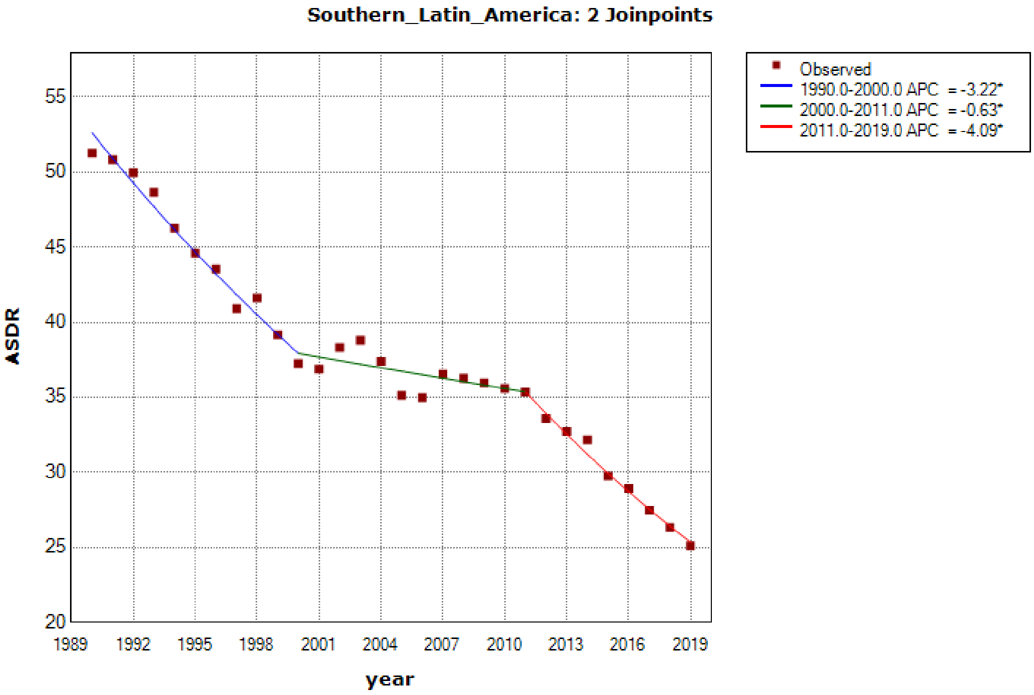

Figure S38 Trends in the age-standardized disability-adjusted life rate (ASDR, per 100,000 population) of $PM_{2.5}$-related preterm birth in Southern Latin America from 1990 to 2019. *P<0.05

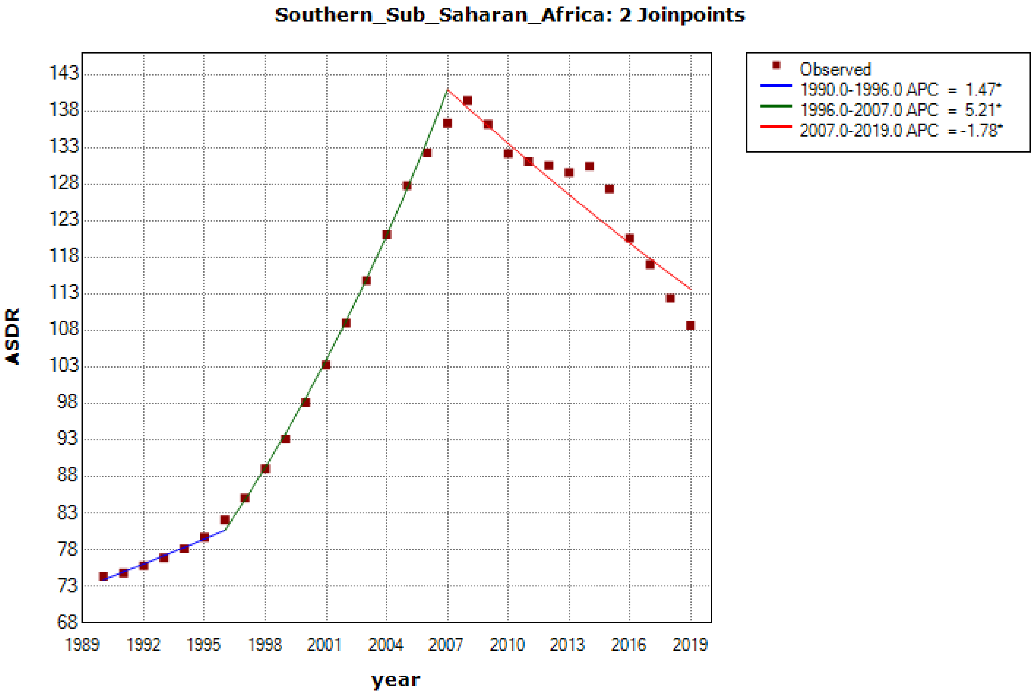

Figure S39 Trends in the age-standardized disability-adjusted life rate (ASDR, per 100,000 population) of $PM_{2.5}$-related preterm birth in Southern Sub Saharan Africa from 1990 to 2019. *P<0.05

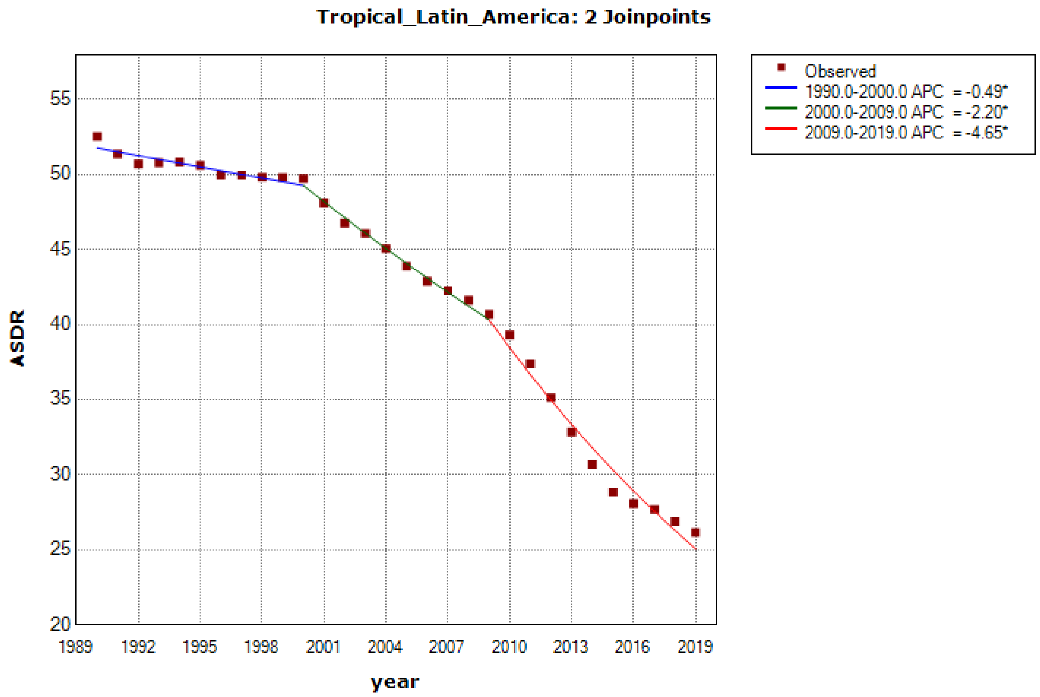

Figure S40 Trends in the age-standardized disability-adjusted life rate (ASDR, per 100,000 population) of $PM_{2.5}$-related preterm birth in Tropical Latin America from 1990 to 2019. $^{*}P<0.05$

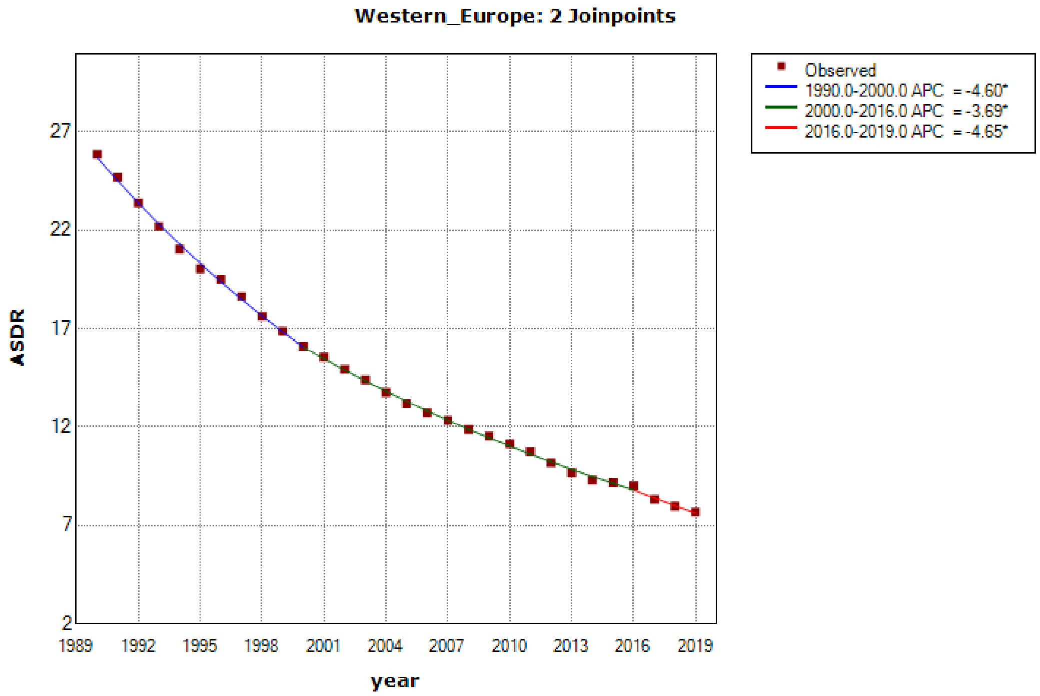

Figure S41 Trends in the age-standardized disability-adjusted life rate (ASDR, per 100,000 population) of $PM_{2.5}$-related preterm birth in Western Europe from 1990 to 2019. $^{*}P<0.05$

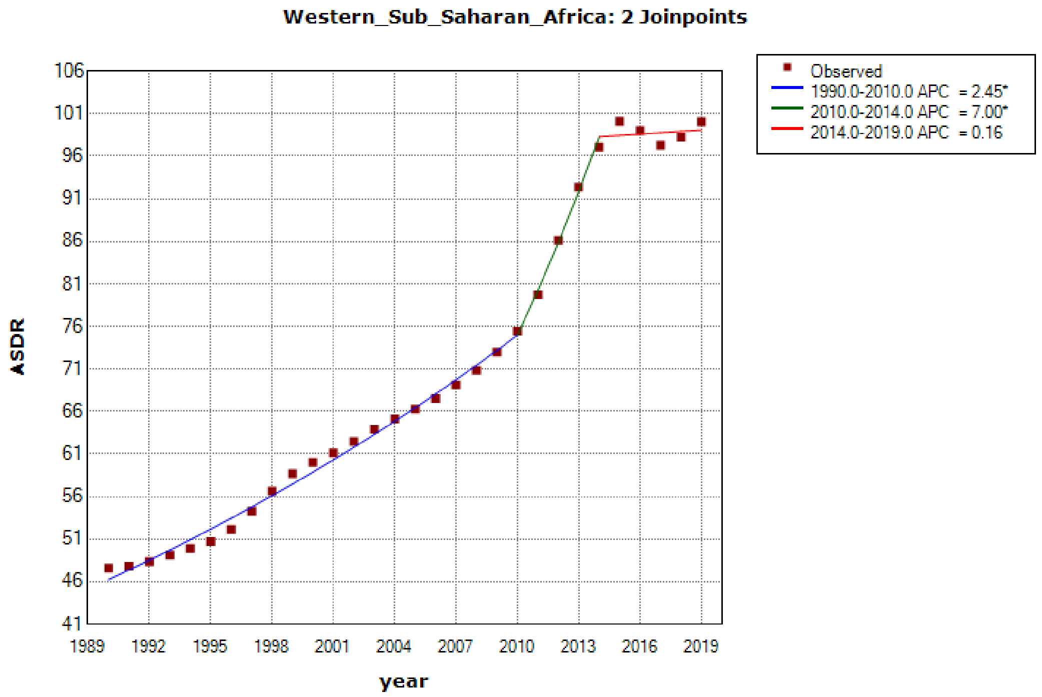

Figure S42 Trends in the age-standardized disability-adjusted life rate (ASDR, per 100,000 population) of $PM_{2.5}$-related preterm birth in Western Sub Saharan Africa from 1990 to 2019. $^{*}P$<0.05
